# RNA-seq reveals the pan-transcriptomic impact of attenuating the gliotoxin self-protection mechanism in *Aspergillus fumigatus*

**DOI:** 10.1186/1471-2164-15-894

**Published:** 2014-10-14

**Authors:** Grainne O’Keeffe, Stephen Hammel, Rebecca A Owens, Thomas M Keane, David A Fitzpatrick, Gary W Jones, Sean Doyle

**Affiliations:** Department of Biology, National University of Ireland Maynooth, Maynooth, Co. Kildare, Ireland; The Wellcome Trust Sanger Institute, Hinxton Cambridge, CB10 1SA UK

**Keywords:** Gliotoxin, RNA-seq, Transcriptome, Secondary metabolism, Fungal proteomics

## Abstract

**Background:**

*Aspergillus fumigatus* produces a number of secondary metabolites, one of which, gliotoxin, has been shown to exhibit anti-fungal activity. Thus, *A. fumigatus* must be able to protect itself against gliotoxin. Indeed one of the genes in the gliotoxin biosynthetic gene cluster in *A. fumigatus*, *gliT*, is required for self-protection against the toxin- however the global self-protection mechanism deployed is unclear. RNA-seq was employed to identify genes differentially regulated upon exposure to gliotoxin in *A. fumigatus* wild-type and *A. fumigatus* ∆*gliT*, a strain that is hypersensitive to gliotoxin.

**Results:**

Deletion of *A. fumigatus gliT* resulted in altered expression of 208 genes (log_2_ fold change of 1.5) when compared to *A. fumigatus* wild-type, of which 175 genes were up-regulated and 33 genes were down-regulated. Expression of 164 genes was differentially regulated (log_2_ fold change of 1.5) in *A. fumigatus* wild-type when exposed to gliotoxin, consisting of 101 genes with up-regulated expression and 63 genes with down-regulated expression. Interestingly, a much larger number of genes, 1700, were found to be differentially regulated (log_2_ fold change of 1.5) in *A. fumigatus* ∆*gliT* when challenged with gliotoxin. These consisted of 508 genes with up-regulated expression, and 1192 genes with down-regulated expression. Functional Catalogue (FunCat) classification of differentially regulated genes revealed an enrichment of genes involved in both primary metabolic functions and secondary metabolism. Specifically, genes involved in gliotoxin biosynthesis, helvolic acid biosynthesis, siderophore-iron transport genes and also nitrogen metabolism genes and ribosome biogenesis genes underwent altered expression. It was confirmed that gliotoxin biosynthesis is induced upon exposure to exogenous gliotoxin, production of unrelated secondary metabolites is attenuated in *A. fumigatus* ∆*gliT*, while quantitative proteomic analysis confirmed disrupted translation in *A. fumigatus* ∆*gliT* challenged with exogenous gliotoxin.

**Conclusions:**

This study presents the first global investigation of the transcriptional response to exogenous gliotoxin in *A. fumigatus* wild-type and the hyper-sensitive strain, ∆*gliT*. Our data highlight the global and extensive affects of exogenous gliotoxin on a sensitive strain devoid of a self-protection mechanism and infer that GliT functionality is required for the optimal biosynthesis of selected secondary metabolites in *A. fumigatus*.

**Electronic supplementary material:**

The online version of this article (doi:10.1186/1471-2164-15-894) contains supplementary material, which is available to authorized users.

## Background

Gliotoxin, a non-ribosomally synthesised peptide produced by *Aspergillus fumigatus* and related fungi, is redox-active, depletes cellular glutathione (GSH), causes DNA damage and protein modification, and is consequently capable of inhibiting mammalian, fungal and bacterial cell growth [1–5]. Conversely, it has also been demonstrated that gliotoxin presence protects against H_2_O_2_-induced oxidative stress in *A. fumigatus* and that gliotoxin can substitute for peroxiredoxin in mammalian cells to protect against similar oxidative stresses [6, 7]. In *A. fumigatus*, gliotoxin biosynthesis is encoded by a 13-gene cluster, *gli*, and it has been demonstrated that gliotoxin effects induction of many genes within the *gli* cluster in a positive feedback manner [2, 8, 9]. Thus, gliotoxin presence induces *gli* cluster activation via *gliZ*, a Zn_2_Cys_6_ binuclear transcription factor, and deletion of *gliZ* abolishes gliotoxin biosynthesis [10]. Moreover, Forseth *et al.* [11] revealed that an additional nine metabolites, dependent on *gliZ* presence, are produced consequent to gliotoxin biosynthetic pathway functionality in *A. fumigatus*.

Gliotoxin exposure has been shown by qRT-PCR to either activate or induce increased expression of all genes in the *gli* cluster, as especially observed for *A. fumigatus* ∆*gliP*, deficient in the non-ribosomal peptide synthetase which mediates cyclo-L-Phe-L-Ser formation [9, 12]. Others have shown induction of *gliG* (a glutathione *S*-transferase), *gliA* (an MFS transporter) and *gliT* upon exposure of *A. fumigatus* wild-type to gliotoxin, by Northern analysis [2]. However, definitive evidence of concomitant increased *de novo* gliotoxin production has not been forthcoming. Relatedly, it has been shown that transformation with *A. fumigatus gliA* confers resistance against exogenous gliotoxin upon *Leptosphaeria maculans* [13] while deletion of *gliA* in *A. fumigatus* renders it less resistant to exogenous gliotoxin [14], and Schrettl *et al.* [2] were the first to demonstrate increased GliT abundance by 2D-PAGE/MALDI-ToF analysis in *A. fumigatus* upon exposure to exogenous gliotoxin. However, within *A. fumigatus*, gliotoxin biosynthesis must be controlled to avoid manifestation of the deleterious affects of this reactive metabolite.

Since the original observations that self-protection against gliotoxin was largely mediated by the enzyme GliT, a gliotoxin oxidoreductase [2, 15], it has subsequently been found that other organisms contain similar enzymes which facilitate self-protection against related epipolythiodioxopiperazines [16, 17]. Indeed, in the bacterium, *Streptomyces clavuligerus*, it has been demonstrated that an oxidoreductase, HlmI, confers self-protection against the disulfide-bridge-containing, non-ribosomal peptide, holomycin [17]. Interestingly, an RNA methyltransferase, Hom12 in *Yersinia ruckeri*, also enables self-protection against holomycin, since Hom12 deletion results in acquisition of a holomycin-sensitive phenotype [16]. This clearly infers that self-protection against redox-active non-ribosomal peptides is a multi-faceted process, yet few studies have attempted molecular dissection of the process. Carberry *et al.* [5] revealed significantly elevated GSH levels in *A. fumigatus* Δ*gliT*, and speculated about exacerbation of gliotoxin toxicity, resulting from formation of the dithiol form of gliotoxin, consequent to this apparent dysregulation in the level of an important cellular reductant. Indeed, the apparent resistance of *Saccharomyces cerevisiae* Δ*gsh1*, which exhibits significantly attenuated GSH levels, to exogenous gliotoxin supported this hypothesis. Interestingly, these authors also observed that *S. cerevisiae* Δ*sod1* and Δ*yap1* were hypersensitive to exogenous gliotoxin, suggesting that a deficient oxidative stress response sensitizes this organism to gliotoxin. Coleman *et al.* [4] further revealed that both *Candida albicans* and *Cryptococcus neoformans* were sensitive to gliotoxin exposure, however apart from an elegant demonstration of membrane damage consequent to gliotoxin exposure, no mechanistic basis of the anti-fungal effect of gliotoxin was forthcoming.

It is somewhat surprising that the concept of self-protection against gliotoxin, in fungi capable of its biosynthesis, has received scant attention since the discovery of gliotoxin in 1936 - given the reactive nature of this disulfide-containing metabolite. However the availability of powerful new technologies such as RNA-seq [18–21], provides us with a tool to address this information deficient. Consequently, we present here the first exploration of the global transcriptomic response of both *A. fumigatus* wild-type and ∆*gliT* to exogenous gliotoxin, which illuminates the important role played by *gliT* in mediating control of the cellular systems in the presence of this reactive metabolite.

## Results

### Deletion of *gliT*results in altered expression of over 200 genes involved in many functions in *A. fumigatus*

**Figure 1:**
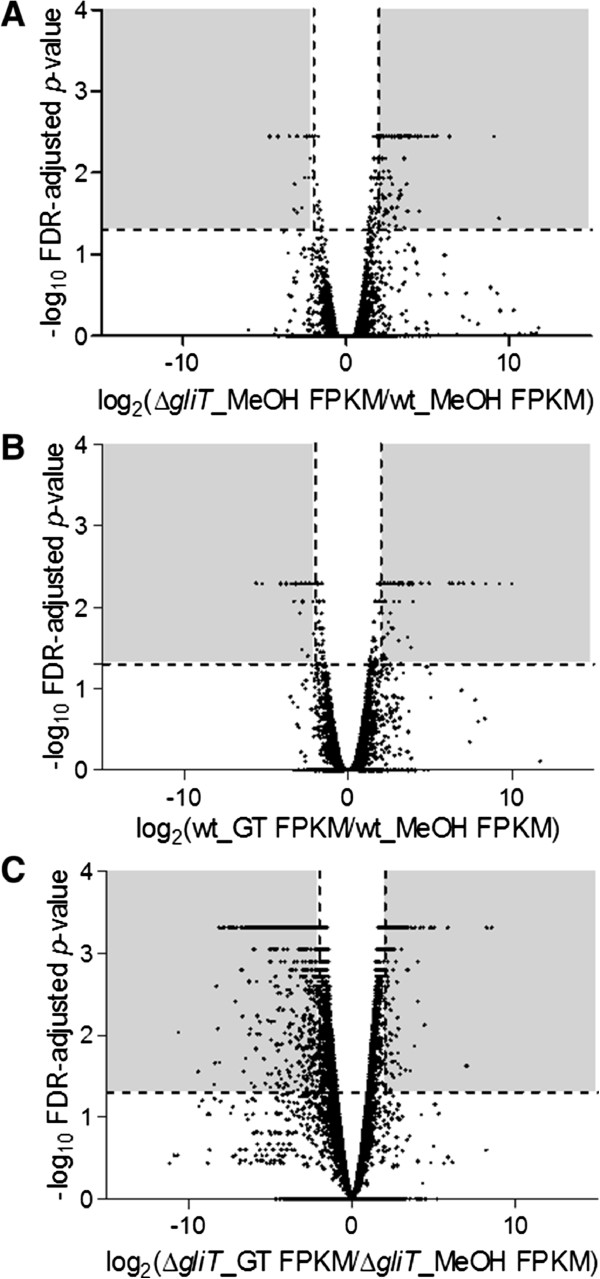
**Overall impact of** ***gliT*** **loss and gliotoxin on** ***A. fumigatus*** **gene expression. A**. Differentially regulated genes between *A. fumigatus* ∆*gliT* methanol control (∆*gliT*_MeOH) and *A. fumigatus* wild-type methanol control (wt-MeOH). The rFPKM value (FPKM (∆*gliT*_MeOH)/FPKM (wt_MeOH) was plotted for each gene. **B**. Differentially regulated genes between *A. fumigatus* wild-type methanol control (wt_MeOH) and *A. fumigatus* wild-type exposed to gliotoxin (wt_GT). The rFPKM value (FPKM (wt_GT)/FPKM (wt_MeOH) was plotted for each gene. **C**. Differentially regulated genes between *A. fumigatus* ∆*gliT* methanol control (∆*gliT*_MeOH) and *A. fumigatus* ∆*gliT* exposed to gliotoxin (∆*gliT*_GT). The rFPKM value (FPKM (∆*gliT*_GT)/FPKM (∆*gliT*_MeOH) was plotted for each gene. The grey shaded areas highlight the significantly (*p* <0.05) up-regulated (log_2_ fold-change ≥2) and down-regulated (log_2_ fold-change ≤2) genes in each set.

**Figure 2:**
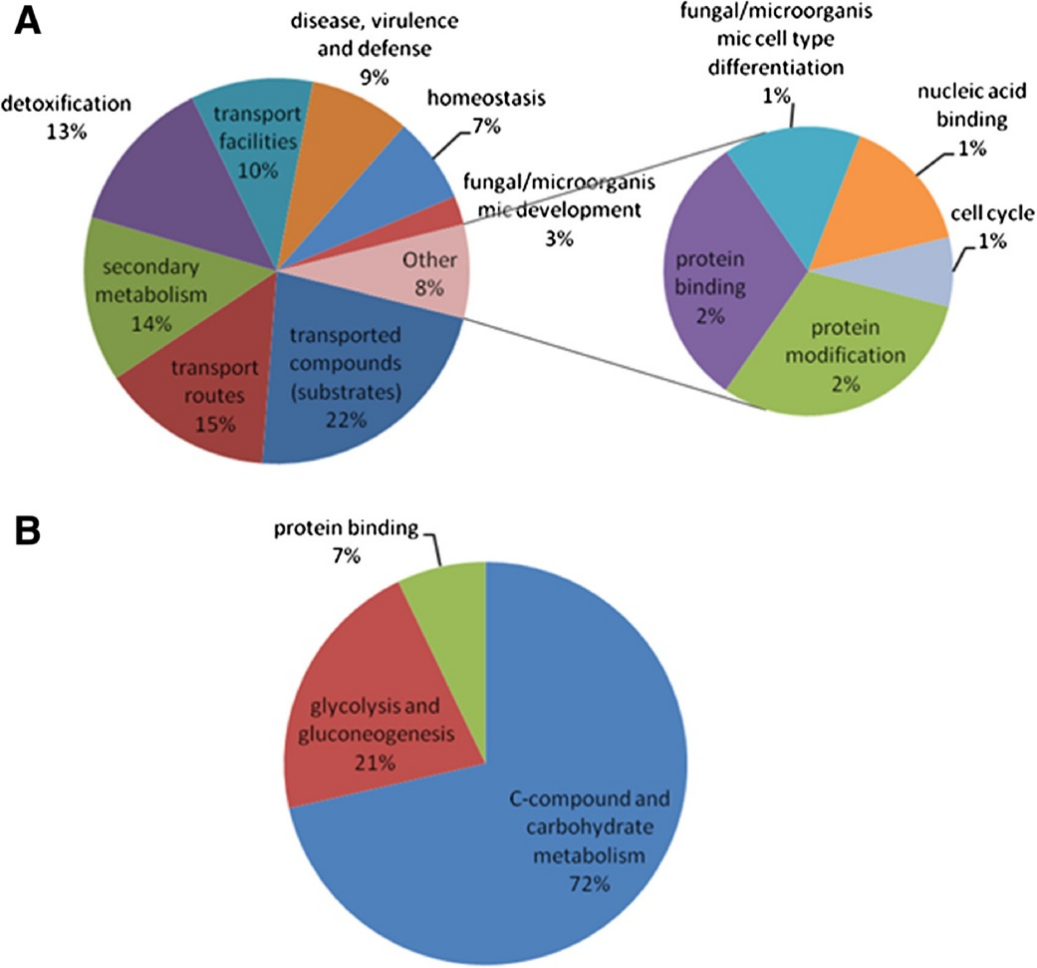
**Functional classification into FunCat 2** ^**nd**^ **level categories of significantly A. up-regulated genes in** ***A. fumigatus*** **∆** ***gliT*** **compared to wild-type and B. down-regulated genes in** ***A. fumigatus*** **∆** ***gliT*** **compared to wild-type.**

As deletion of *A. fumigatus gliT* renders the strain sensitive to exogenous gliotoxin [2, 15], to achieve a better understanding of the self-protection against gliotoxin provided by *A. fumigatus gliT*, high throughput RNA sequencing analysis was carried out. An average of 9312 transcripts were expressed in *A. fumigatus* wild-type and ∆*gliT* (available from the European Nucleotide Archive under accession ERP001382), which is in accordance with other RNA-seq investigations of the *A. fumigatus* transcriptome [18, 22]. A comparison of *A. fumigatus* wild-type and ∆*gliT* revealed that the deletion of *gliT* resulted in the significant (*p* < 0.05) dysregulated expression of 208 genes, consisting of 175 up-regulated genes while 33 genes were significantly down-regulated (*p* < 0.05) (Figure 1A, Additional file 1). The differentially regulated genes were classified according to the Functional Catalogue (FunCat) [23] and KEGG pathways [24] to identify the functions of these genes. Following deletion of *gliT*, 13 2^nd^ level, 19 3^rd^ level and 12 4^th^ level FunCat categories were over-represented in the up-regulated gene set, compared to 3 2^nd^ level, 10 3^rd^ level and 3 4^th^ level FunCat categories enriched in the down-regulated gene set (Additional file 2: Tables S1 and S2). Categories over-represented in the up-regulated gene set include; secondary metabolism, transport and detoxification, while genes involved in C-compound and carbohydrate metabolism were enriched in the down-regulated gene set (Figure 2). Additionally, 11 KEGG pathways were over-represented in the up-regulated gene set, while genes involved in 13 KEGG pathways were enriched in the down-regulated gene set (Additional file 2: Tables S3 and S4). Carbohydrate and lipid metabolism were over-represented in the up-regulated gene set, while amino acid metabolism was enriched in the down-regulated gene set. Genes involved in secondary metabolite biosynthesis were enriched in both the up- and down-regulated gene sets.

### Gliotoxin exposure alters the regulation of hundreds of genes in both *A. fumigatus*wild-type and ∆*gliT*

Following *A. fumigatus* wild-type exposure to exogenous gliotoxin, 164 genes were significantly differentially regulated (*p* <0.05) compared to control conditions (Figure 1B). Of these 164 genes, 101 genes were up-regulated when *A. fumigatus* wild-type was exposed to gliotoxin, while 63 genes were down-regulated (Additional file 1). In contrast, when *A. fumigatus* ∆*gliT* was exposed to exogenous gliotoxin, 1,700 genes were significantly differentially regulated (*p* <0.05) (Figure 1C), whereby expression of 508 genes was up-regulated in response to gliotoxin exposure and 1192 genes down-regulated (Additional file 1). Given the large transcriptomic remodelling observed in *A. fumigatus* ∆*gliT* following exogenous gliotoxin exposure, cell viability was assessed at 85%. Although a significant decrease (*p* =0.0019) in the cell viability of *A. fumigatus* ∆*gliT* exposed to exogenous gliotoxin, compared with the methanol control (97%) (Additional file 2: Figure S1), gross cell death was not observed.

### Functional characterisation of differentially regulated genes in *A. fumigatus*wild-type and ∆*gliT*following exogenous gliotoxin exposure

**Figure 3:**
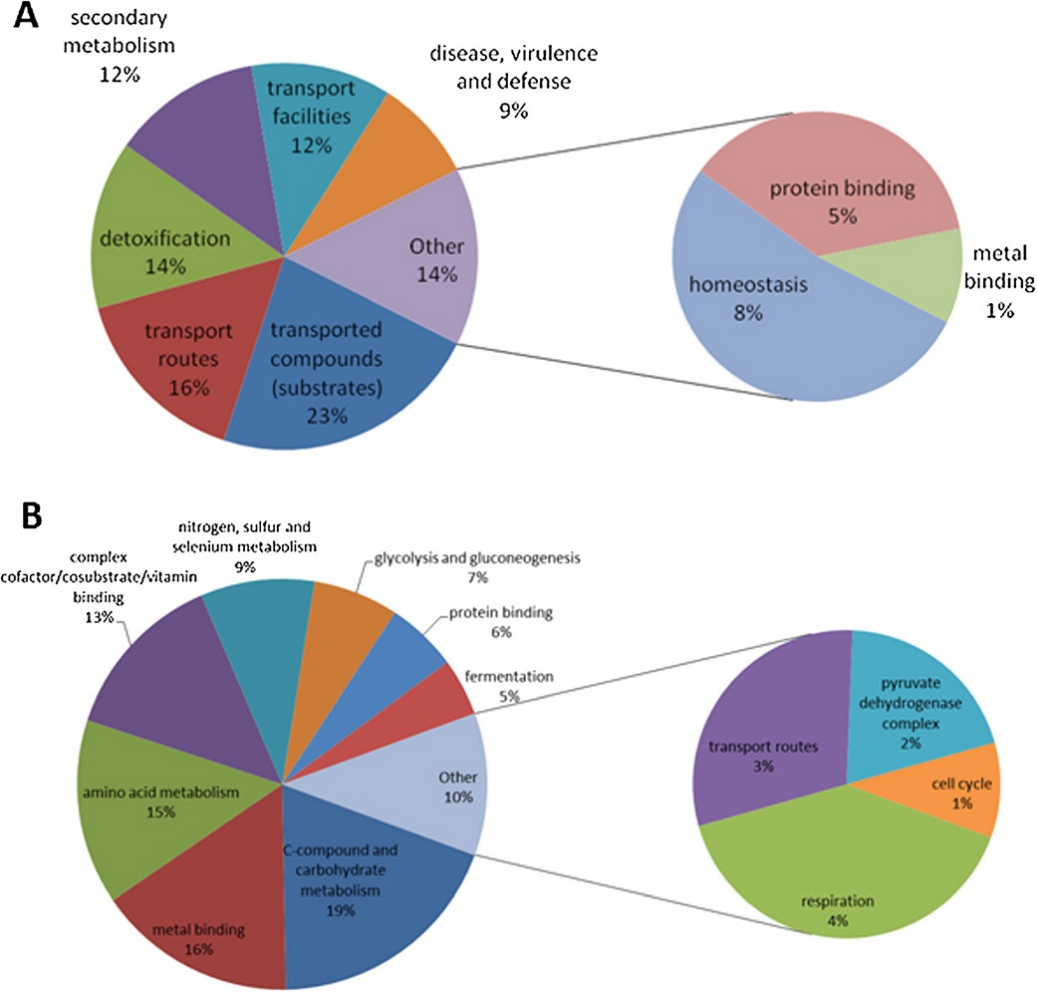
**Functional classification into FunCat 2** ^**nd**^ **level categories of significantly A. up-regulated genes in** ***A. fumigatus*** **wild-type exposed to gliotoxin compared to the MeOH control and B. down-regulated genes in** ***A. fumigatus*** **wild-type exposed to gliotoxin compared to the MeOH control.**

**Figure 4:**
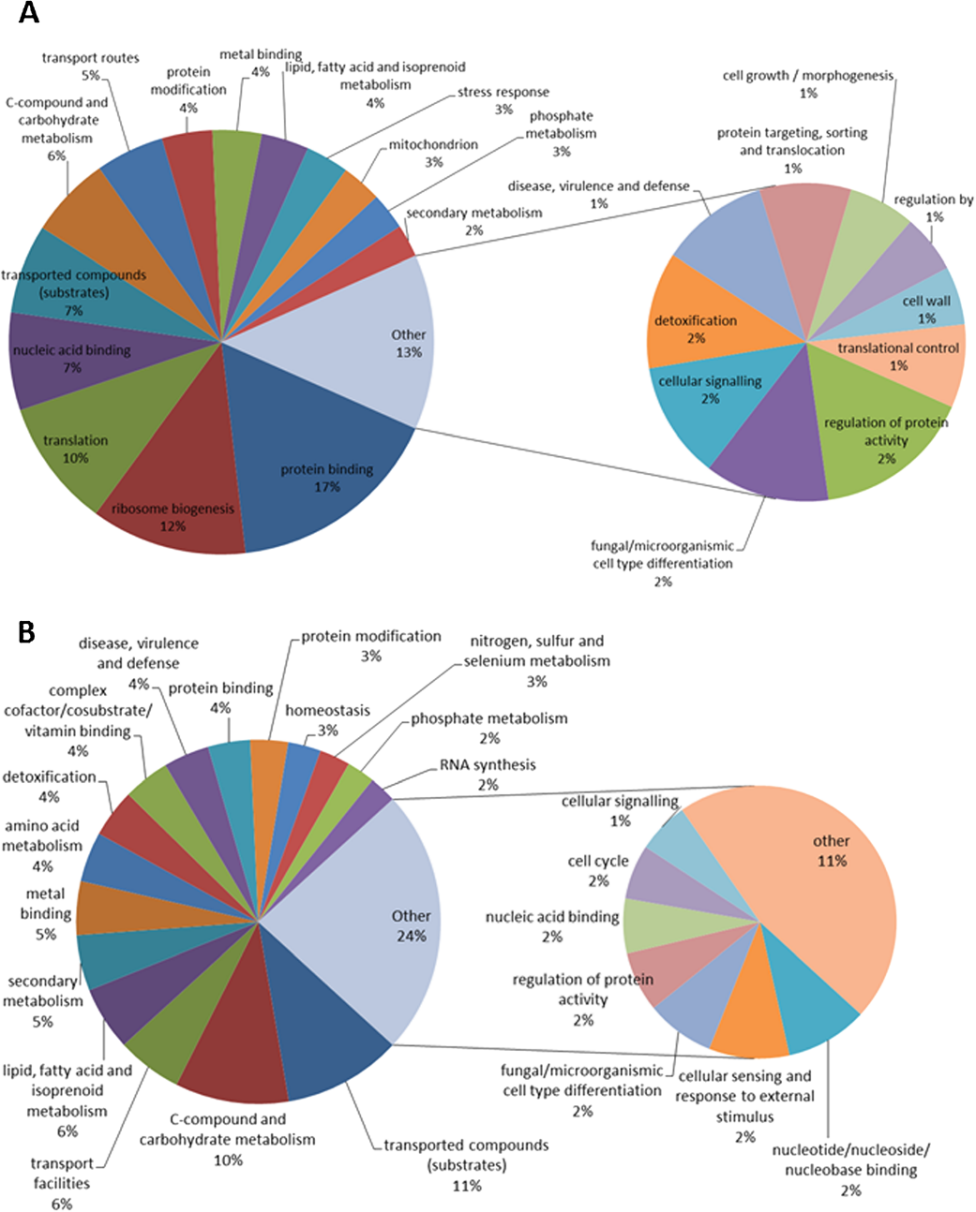
**Functional classification into FunCat 2** ^**nd**^ **level categories of significantly A. up-regulated genes in** ***A. fumigatus*** **∆** ***gliT*** **exposed to gliotoxin compared to the MeOH control and B. down-regulated genes in** ***A. fumigatus*** **∆** ***gliT*** **exposed to gliotoxin compared to the MeOH control.**

In classifying the differentially regulated genes in *A. fumigatus* wild-type upon exposure to exogenous gliotoxin, 9 2^nd^ level, 17 3^rd^ level and 11 4^th^ level FunCat categories, respectively, were over-represented in the up-regulated gene set compared to 12 2^nd^ level, 17 3^rd^ level and 8 4^th^ level categories in the down-regulated gene set (Additional file 2: Tables S5 and S6). Comparatively, in *A. fumigatus* ∆*gliT* exposed to exogenous gliotoxin, a greater number of FunCat categories were over-represented. Here, 24 2^nd^ level, 23 3^rd^ level and 12 4^th^ level FunCat categories, respectively, were over-represented in the up-regulated gene set compared to 42 2^nd^ level, 84 3^rd^ level and 39 4^th^ level categories in the down-regulated gene set (Additional file 2: Tables S7 and S8). *A. fumigatus* wild-type exposure to exogenous gliotoxin results in an over-representation of up-regulated expression of genes involved in secondary metabolism, transport (particularly siderophore-iron transport), detoxification processes and homeostasis processes as well as others (Figure 3). Of the down-regulated genes, some of the categories which were enriched included amino acid metabolism, carbohydrate metabolism and complex cofactor/cosubstrate/vitamin binding (Figure 3). Loss of *gliT* in combination with exogenous gliotoxin exposure results in the dysregulation of a large number of processes within the cell. It resulted in an over-representation of up-regulated genes involved in stress response, ribosome biogenesis and translation, and of down-regulated genes involved in metabolism of cysteine, nitrogen, sulphur and selenium metabolism, RNA synthesis, transport (including siderophore-iron transport), homeostasis and cellular import (Figure 4).

In combination with the identified FunCat categories, 2 KEGG pathways were over-represented in the up-regulated gene set, and 13 KEGG pathways in the down-regulated gene set of *A. fumigatus* wild-type upon exogenous gliotoxin exposure were over-represented (Additional file 2: Tables S9 and S10). Pathways involved in xenobiotic biodegradation and metabolism were enriched in the up-regulated gene set, while in the down-regulated gene set, pathways involved in the biosynthesis of secondary metabolites, glycolysis/gluconeogenesis and amino acid metabolism are over-represented. In *A. fumigatus* ∆*gliT* exposed to exogenous gliotoxin, 18 KEGG pathways were over-represented in the up-regulated gene set compared to 38 KEGG pathways in the down-regulated gene set (Additional file 2: Tables S11 and S12). Among the KEGG pathways over-represented in the up-regulated gene set were pathways involved in carbohydrate metabolism, translation and selenoamino acid metabolism. In the down-regulated gene set, some of the pathways over-represented included; biosynthesis of secondary metabolites, starch and sucrose metabolism, and amino acid metabolism.

### Exogenous gliotoxin causes dysregulation of gliotoxin biosynthesis cluster gene expression in both *A. fumigatus*wild-type and ∆*gliT*

Close inspection of the 13-gene gliotoxin biosynthesis cluster [2, 3], revealed that exogenous gliotoxin caused the dysregulated expression of a number of gliotoxin biosynthetic genes in both *A. fumigatus* wild-type and ∆*gliT* (Table 1). Upon exposure to exogenous gliotoxin, five out of the 13 genes in the cluster were significantly up-regulated in *A. fumigatus* wild-type. Expression of *A. fumigatus gliZ* was up-regulated log_2_ 3.2-fold, *gliP* log_2_ 6.2 fold, while *gliA* and *gliF* were up-regulated log_2_ 10- and log_2_ 6.7-fold, respectively. Expression of *gliT*, which confers protection against exogenous gliotoxin [2, 15], was up-regulated log_2_ 9.2-fold. Increased expression of the remaining genes in the gliotoxin biosynthetic cluster was also observed in *A. fumigatus* wild-type upon exogenous gliotoxin exposure, however altered expression was not significant.

**Table 1:** **Log**_**2**_ **(fold change) in the expression of the gliotoxin biosynthetic genes in** ***A. fumigatus*** **wild-type and ∆** ***gliT*** **exposed to exogenous gliotoxin**

		Wild-type_Glio v Wild-type_MeOH		∆***gliT***_Glio v ∆***gliT***_MeOH	
Gene	Gene name	Log_2_(fold change)	q value	Log_2_(fold change)	q value
AFUA_6G09630	*gliZ*	3.165	0.005	0.465	0.525
AFUA_6G09640	*gliI*	1.758	1.000	−0.031	1.000
AFUA_6G09650	*gliJ*	2.250	0.383	−2.816	1.000
AFUA_6G09660	*gliP*	6.208	0.005	3.408	0.0005
AFUA_6G09670	*gliC*	5.023	0.065	4.223	0.093
AFUA_6G09680	*gliM*	11.737	0.769	6.986	0.024
AFUA_6G09690	*gliG*	7.956	0.253	8.199	0.254
AFUA_6G09700	*gliK*	7.417	0.445	2.844	0.073
AFUA_6G09710	*gliA*	10.014	0.005	8.517	0.0005
AFUA_6G09720	*gliN*	4.807	0.053	2.106	0.086
AFUA_6G09730	*gliF*	6.758	0.005	4.745	0.0005
AFUA_6G09740	*gliT*	9.248	0.005	-	-
AFUA_6G09745	*gliH*	5.075	0.128	2.688	0.237

**Figure 5:**
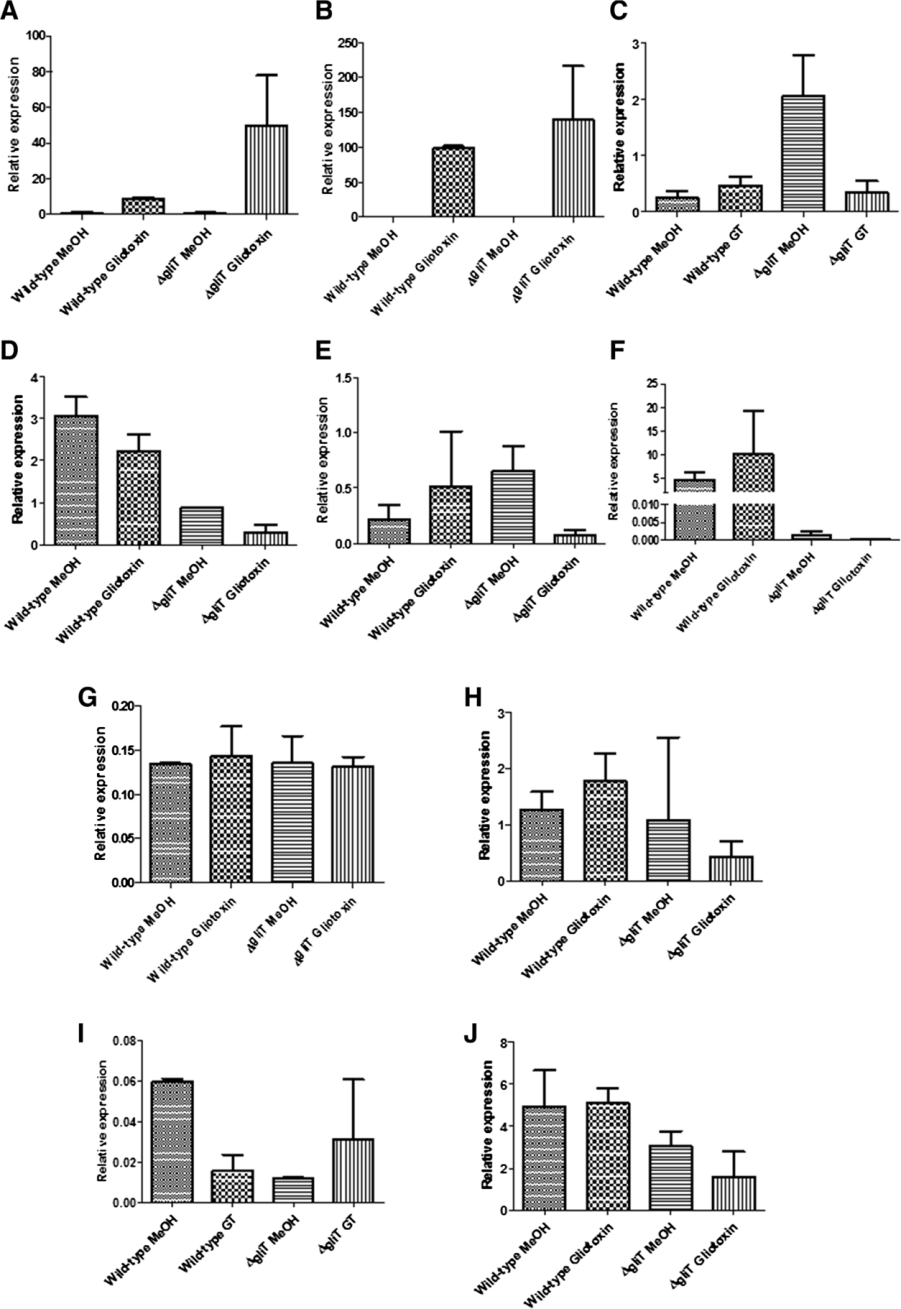
**qRT-PCR analysis of** ***A. fumigatus gliZ*** **(A)** ***, gliA*** **(B)** ***, osc3*** **(C),** ***ftmA*** **(D),** ***fma-pks*** **(E),** ***psoA*** **(F),** ***laeA*** **(G), AFUA_3G13700 (H),** ***sidH*** **(I) and** ***optB*** **(J) expression in** ***A. fumigatus*** **wild-type and** ***A. fumigatus*** **∆** ***gliT*** **in the presence and absence of exogenous gliotoxin.**

Upon *A. fumigatus* ∆*gliT* exposure to exogenous gliotoxin, dysregulated expression of gliotoxin biosynthetic genes was also observed (Table 1). As in *A. fumigatus* wild-type, *gliP, gliA* and *gliF* expression was also up-regulated in ∆*gliT* upon exogenous gliotoxin addition. *A. fumigatus gliP* was up-regulated log_2_ 3.4-fold, *gliA* was up-regulated log_2_ 8.5-fold and *A. fumigatus gliF* was up-regulated log_2_ 4.7-fold. Additionally, *A. fumigatus gliM* was up-regulated log_2_ 6.9-fold in *A. fumigatus* ∆*gliT* exposed to exogenous gliotoxin, which was not observed in *A. fumigatus* wild-type upon gliotoxin exposure. Of the remaining gliotoxin biosynthetic genes, while not significant, with the exception of *A. fumigatus gliZ* and *gliI* which did not exhibit altered expression, and *gliJ* which appeared to undergo down-regulated expression, increased expression of the remaining genes was observed in *A. fumigatus* ∆*gliT* upon exposure to exogenous gliotoxin. Quantitative real-time PCR (qRT-PCR) analysis of the expression of *A. fumigatus gliZ* confirmed the up-regulation in *A. fumigatus* wild-type exposed to exogenous gliotoxin compared to the control, however it also showed increased *gliZ* expression in ∆*gliT* in response to gliotoxin (Figure 5), which was not observed in the RNA-seq analysis, possibly due to the sensitivities of the different techniques. qRT-PCR analysis of the expression of *A. fumigatus gliA* in both *A. fumigatus* wild-type and *A. fumigatus* ∆*gliT* in both the absence and presence of exogenous gliotoxin confirmed the up-regulation in response to exogenous gliotoxin (Figure 5).

**Figure 6:**
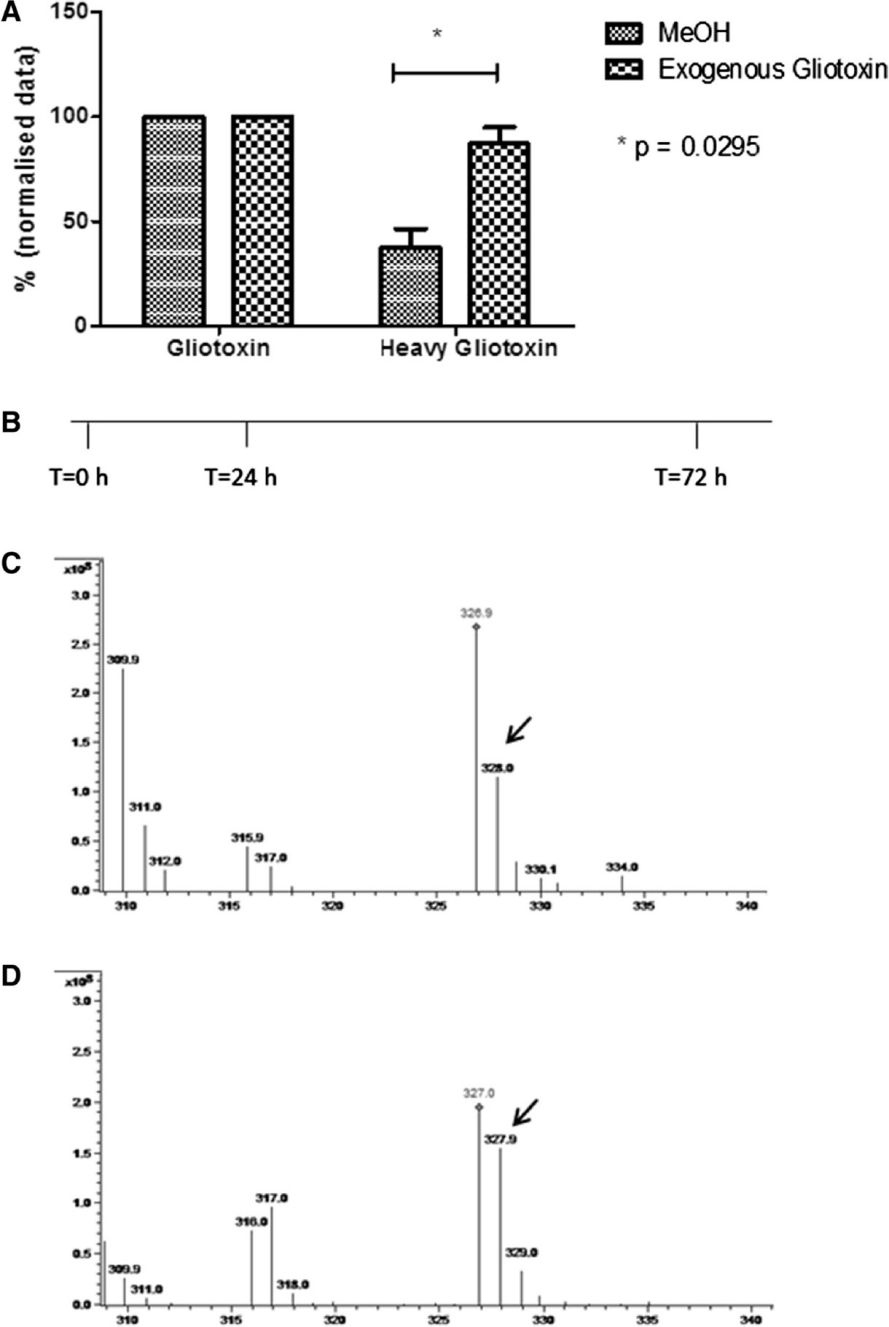
**Gliotoxin biosynthesis is induced with exogenous gliotoxin addition. A.** A significant increase (*p* =0.0295) in the amount of *de novo* gliotoxin ([^13^C]-gliotoxin) produced following addition of exogenous gliotoxin was observed compared to the methanol control. **B.** Time-line of the experimental conditions. Cultures (duplicate) were inoculated at T =0 h. At T =24 h, [^13^C]-phenylalanine was added along with gliotoxin (5 μg/ml final) or methanol (solvent control). Culture supernatants were collected at T =72 h and organic extraction carried out prior to LC-MS/MS analysis. **C.** Spectrum showing gliotoxin (*m/z* 327) and [^13^C]-gliotoxin (*m/z* 328) in cultures spiked with the methanol. **D.** Spectrum showing gliotoxin (*m/z* 327) and [^13^C]-gliotoxin (*m/z* 328) in cultures spiked with exogenous gliotoxin. The black arrows highlight [^13^C]-gliotoxin, where higher levels are observed in cultures induced by exogenous gliotoxin compared to the methanol control.

The impact of gliotoxin on the expression of the gliotoxin biosynthetic genes is in accordance with that observed in other studies using different techniques [2, 9]. Moreover, feeding experiments with [^13^C]-phenylalanine herein confirm, for the first time, that gliotoxin biosynthesis is actually induced by exogenous gliotoxin. A significant increase (*p* =0.0295) was observed in the amount of [^13^C]-gliotoxin detectable in the wild-type *A. fumigatus* Af293 culture supernatants following exogenous gliotoxin addition compared to the methanol control (Figure 6).

### Exogenous gliotoxin results in down-regulation of secondary metabolite gene cluster expression in *A. fumigatus*∆*gliT*

FunCat classification of the altered gene expression in *A. fumigatus* wild-type and ∆*gliT*, respectively, revealed an enrichment of genes involved in secondary metabolism upon exogenous gliotoxin exposure. In *A. fumigatus* wild-type, expression of 16 genes which was up-regulated, and that of 3 genes down-regulated in response to exogenous gliotoxin, were classified by 2^nd^ level of FunCat as being involved in secondary metabolism (Additional file 2: Tables S5 and S6). A larger number of genes were classified in this category in *A. fumigatus* ∆*gliT* in response to exogenous gliotoxin presence, where 22 up-regulated genes and 103 down-regulated genes were observed (Additional file 2: Tables S7 and S8). A closer inspection of some of the secondary metabolite genes clusters revealed significant alterations in the expression of genes in the helvolic biosynthesis cluster [25] and the “supercluster” on chromosome 8 which encodes the biosynthetic pathways of a number of secondary metabolites, including fumitremorgin B, fumagillin and pseurotin A [26–29] (Table 2).

**Table 2:** **Log**_**2**_ **(fold change) in helvolic acid, fumitremorgin, fumagillin and pseurotin A biosynthetic gene cluster expression in** ***A. fumigatus*** **wild-type and ∆** ***gliT*** **exposed to exogenous gliotoxin**

		Wild-type_Glio v Wild-type_MeOH		∆***gliT***_Glio v ∆***gliT***_MeOH	
Gene	Gene name	Log_2_(fold change)	q value	Log_2_(fold change)	q value
**Helvolic acid**					
AFUA_4G14770	*osc3*	3.279	0.005	−6.296	0.010
AFUA_4G14780	*cyp5081A1*	2.425	0.076	−6.515	0.092
AFUA_4G14790	*cyp5081B1*	1.993	0.228	Absent^a^	0.0005
AFUA_4G14800	*sdr1*	3.219	0.049	−7.163	0.293
AFUA_4G14810	*cyp5081D1*	2.633	0.200	−3.952	0.053
AFUA_4G14820	*-*	2.701	0.057	−5.964	0.063
AFUA_4G14830	*cyp5081C1*	0.470	1.000	−4.229	1.000
AFUA_4G14840	*-*	1.542	1.000	−2.941	1.000
AFUA_4G14850	-	0.960	0.761	−2.702	0.021
**Fumitremorgin B**					
AFUA_8G00170	*ftmA*	−2.777	0.008	−6.649	0.028
AFUA_8G00190	*ftmC*	−1.278	0.205	−5.681	0.0005
AFUA_8G00200	*ftmD*	−1.263	0.181	−5.870	0.0005
AFUA_8G00210	*ftmPT1*	−1.456	0.310	−8.843	0.092
AFUA_8G00220	*ftmE*	−0.614	1.000	Absent^a^	0.0005
AFUA_8G00230	*ftmF*	−0.474	0.901	−10.867	0.293
AFUA_8G00240	*ftmG*	0.611	0.844	−6.519	0.019
AFUA_8G00250	*ftmPT2*	0.581	0.844	−7.300	0.074
AFUA_8G00260	*ftmI*	0.031	0.997	−1.455	0.039
**Fumagillin**					
AFUA_8G00370	*fma-PKS*	0.396	0.946	−7.287	0.0005
AFUA_8G00380	*fma-AT*	0.264	0.968	−9.364	0.074
AFUA_8G00390	*-*	0.220	0.976	−7.902	0.0005
AFUA_8G00400	*-*	−0.025	0.997	−8.094	0.022
AFUA_8G00410	*metAP/fpaII*	−0.113	0.987	−5.196	0.0005
AFUA_8G00420	*fapR/fumR*	−0.284	0.957	−5.826	0.0005
AFUA_8G00430	*-*	0.265	0.969	−7.093	0.0005
AFUA_8G00440	*psoF*	0.005	0.998	−5.426	0.0005
AFUA_8G00460	*fpaI*	−0.301	0.919	−0.282	0.633
AFUA_8G00470	*fmaE*	−0.176	0.968	−0.271	0.636
AFUA_8G00480	*fmaF*	0.427	0.919	−7.258	0.0005
AFUA_8G00490	*-*	0.306	0.959	−5.649	0.007
AFUA_8G00500	*-*	−0.047	0.997	−6.780	0.002
AFUA_8G00510	*fmaG*	0.793	0.820	−8.101	0.0005
AFUA_8G00520	*fma-TC/fmaA*	0.528	0.904	−8.369	0.022
**Pseurotin A**					
AFUA_8G00530	*psoB*	0.415	0.946	−7.450	0.0005
AFUA_8G00540	*psoA/NRPS14*	0.768	0.776	−5.792	0.0005
AFUA_8G00550	*psoC*	0.292	0.964	−7.413	0.0005
AFUA_8G00560	*psoD*	0.808	0.735	−6.778	0.0005
AFUA_8G00570	*-*	−0.271	0.965	−8.059	0.064
AFUA_8G00580	*psoE*	0.306	0.964	−8.206	0.008
AFUA_8G00590	-	−0.982	0.239	−1.430	0.009

^a^Gene expression absent in ∆*gliT*_Glio.

Helvolic acid, a triterpene, is encoded by a 9-gene cluster on chromosome 4 [25]. In *A. fumigatus* wild-type, exogenous gliotoxin results in the significant up-regulation of 2 genes, *A. fumigatus osc3* (log_2_ 3.28-fold) and *sdrI* (log_2_ 3.22-fold) (Table 2). Conversely, in *A. fumigatus* ∆*gliT* exposed to exogenous gliotoxin, *osc3* was significantly down-regulated (log_2_ 6.3-fold) along with a predicted *O*-methyltransferase, AFUA_4G14580, which is significantly down-regulated (log_2_ 2.7-fold) (Table 2). In addition to this, expression of *cyp5081B1* is completely abrogated in *A. fumigatus* ∆*gliT* exposed to exogenous gliotoxin (Table 2). qRT-PCR analysis of *A. fumigatus osc3* confirmed the observed down-regulation in *A. fumigatus* ∆*gliT* and up-regulation of *A. fumigatus osc3* in *A. fumigatus* wild-type exposed to exogenous gliotoxin (Figure 5).

**Figure 7:**
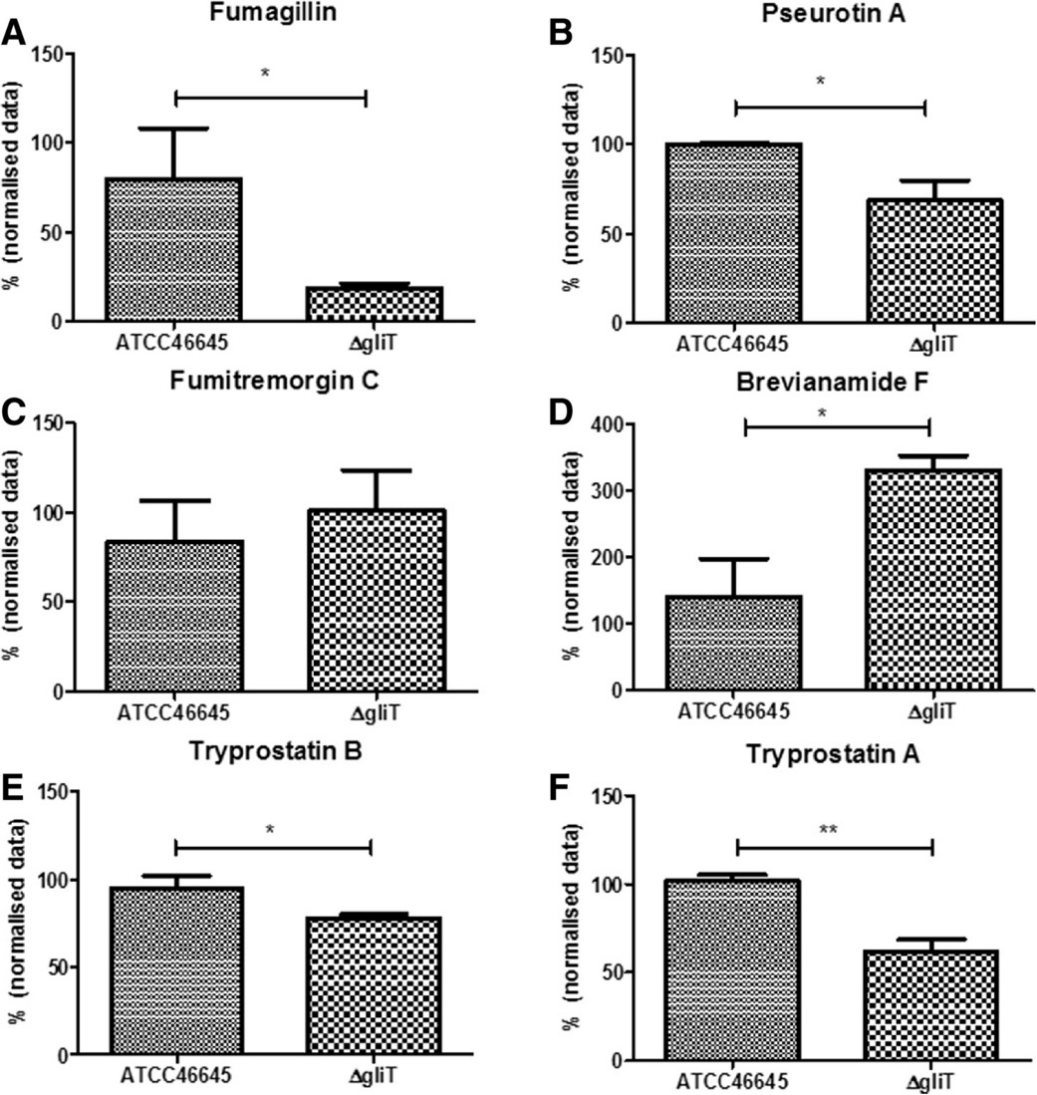
**Levels of fumagillin, pseurotin A, fumitremorgin C, tryprostatin B and tryprostatin A in** ***A. fumigatus*** **wild-type and ∆** ***gliT*** **following 96 h growth under secondary metabolite inducing conditions.** Fumagillin **(A)** and pseurotin A **(B)** production was significantly reduced in *A. fumigatus* ∆*gliT* compared to wild-type (*p =0.0471 and * *p* =0.0297, respectively). There was no significant difference in fumitremorgin C **(C)** levels, while brevianamide F **(D)** levels were significantly increased (* *p* =0.0243) in ∆*gliT* compared to wild-type. Tryprostatin B **(E)** and tryprostatin A **(F)** levels were significantly reduced in ∆*gliT* compared to wild-type (* *p* =0.0453 and ** *p* =0.008, respectively).

Of the 69 genes in the “supercluster” on chromosome 8, expression of two genes is significantly down-regulated in *A. fumigatus* wild-type in response to exogenous gliotoxin (Table 2). However, in *A. fumigatus* ∆*gliT*, when exposed to exogenous gliotoxin, expression of 26 genes from the “supercluster” was down-regulated (Table 2). Closer inspection of the fumitremorgin B biosynthetic genes revealed that expression of the non-ribosomal peptide synthetase (NRPS), *A. fumigatus ftmA* [29], was significantly down-regulated (log_2_ 2.78-fold) in *A. fumigatus* wild-type in exogenous gliotoxin presence (Table 2). While in *A. fumigatus* ∆*gliT*, *ftmA* (log_2_ 6.65-fold)*, ftmC* (log_2_ 5.68-fold)*, ftmD* (log_2_ 5.87-fold)*, ftmG* (log_2_ 6.52-fold) and *ftmI* (log_2_ 1.45-fold) expression was significantly down-regulated, and *ftmE* expression was completely inhibited in response to exogenous gliotoxin (Table 2). qRT-PCR analysis of *A. fumigatus ftmA* confirmed decreased expression in both wild-type and ∆*gliT* following exposure to exogenous gliotoxin (Figure 5). Determination of the levels of the fumitremorgins and associated compounds in *A. fumigatus* wild-type and ∆*gliT* cultured in secondary metabolite-inducing conditions (96 h in Czpaeks-Dox Broth) revealed significant alterations in the levels of a number of cognate metabolites. Specifically, brevianamide F levels were significantly increased in *A. fumigatus* ∆*gliT* compared to wild-type (*p* =0.0243), while the levels of both tryprostatin A and tryprostatin B were significantly decreased (*p* =0.008 and *p* =0.0453) in *A. fumigatus* ∆*gliT* compared to wild-type (Figure 7). There was no significant difference determined in the level of fumitremorgin C between *A. fumigatus* wild-type and ∆*gliT* (Figure 7).

There was no significant dysregulation of the fumagillin biosynthetic genes in *A. fumigatus* wild-type in exogenous gliotoxin presence. However, when *A. fumigatus* ∆*gliT* was exposed to exogenous gliotoxin, expression of 12 fumagillin biosynthetic genes in the cluster was significantly down-regulated (*p* <0.05) (Table 2). Some of the down-regulated genes include a polyketide synthase (PKS) (*A. fumigatus fma-PKS*) [30] and a putative PKS (AFUA_8G00490) [27] which were down-regulated log_2_ 7.20- and log_2_ 5.65-fold, respectively, *A. fumigatus metAP/fpaII* expression was down-regulated log_2_ 5.20-fold and *fma-TC/fmaA* was down-regulated log_2_ 8.37-fold. The decreased expression of *A. fumigatus fma-PKS* in *A. fumigatus* ∆*gliT* exposed to exogenous gliotoxin was confirmed by qRT-PCR analysis (Figure 5). As was the case for fumitremorgin B and fumagillin, none of the pseurotin A biosynthetic genes were differentially regulated in *A. fumigatus* wild-type in exogenous gliotoxin presence (Table 2). However, in *A. fumigatus* ∆*gliT* exposed to exogenous gliotoxin, with the exception of AFUA_8G00570, expression of all of the pseurotin A biosynthetic genes was significantly down-regulated (Table 2). Expression of AFUA_8G00530 and *A. fumigatus psoA/nrps14*, the PKS-NRPS hybrid [28], were down-regulated log_2_ 7.45- and log_2_ 5.79-fold respectively, while *A. fumigatus psoC, psoD* and *elfB* [31] expression was down-regulated log_2_ 7.41-, log_2_ 6.78- and log_2_ 8.21-fold respectively. qRT-PCR confirmed the decreased expression of *A. fumigatus psoA/nrps14* in *A. fumigatus* ∆*gliT* upon exogenous gliotoxin exposure (Figure 5). Relevantly, under secondary metabolite inducing growth conditions, production of fumagillin and pseurotin A was significantly reduced (*p* =0.0471 and *p* =0.0297, respectively) in *A. fumigatus* ∆*gliT* compared to wild-type (Figure 7).

**Figure 8:**
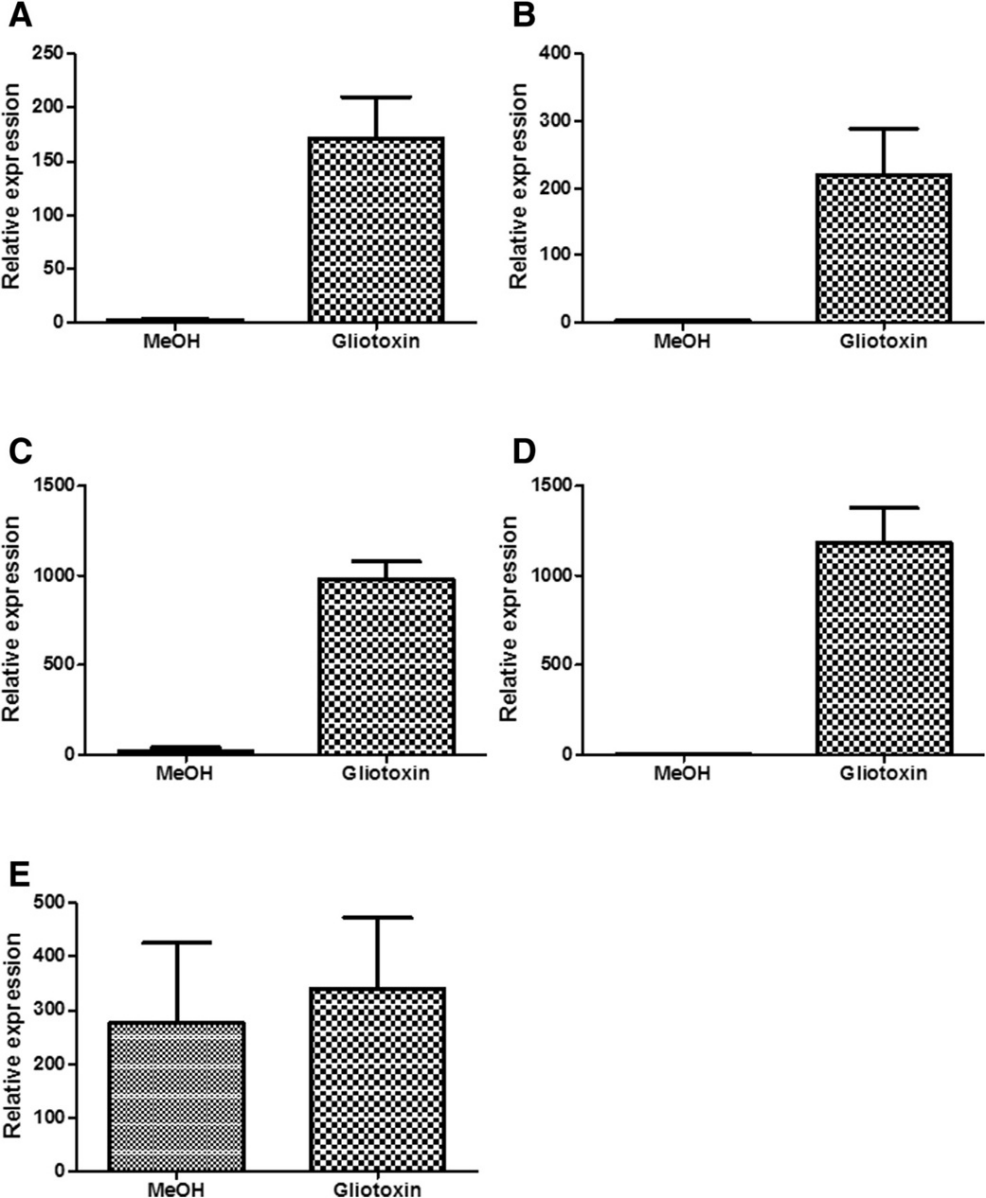
**qRT-PCR analysis of** ***A. fumigatus gliT*** **expression in** ***A. fumigatus*** **∆** ***fmaA*** **(A), ∆** ***psoA*** **(B), ∆** ***laeA*** **(C), ∆** ***fapR*** **(D) and ∆** ***veA*** **(E) exposed to exogenous gliotoxin.**

The methyltransferase, *laeA*, is a global regulator of secondary metabolism in *A. fumigatus*, which fully or partially regulates expression of multiple secondary metabolite gene clusters, including those encoding gliotoxin, helvolic acid, pseurotin A, fumagillin and fumitremorgin biosynthesis [26, 27, 32, 33]. Although dysregulation of secondary metabolite gene expression in *A. fumigatus* ∆*gliT* compared to *A. fumigatus* was observed after exposure to gliotoxin, *laeA* expression was not differentially regulated in *A. fumigatus* wild-type in exogenous gliotoxin presence, but was significantly down-regulated (*p* =0.015; log_2_1.31-fold) in *A. fumigatus* ∆*gliT* under identical conditions. Although significantly down-regulated, it was outside the cut-off of log_2_1.5-fold change. qRT-PCR analysis for *A. fumigatus laeA* did not show any differential regulation in either *A. fumigatus* wild-type or *A. fumigatus* ∆*gliT* when challenged with exogenous gliotoxin (Figure 5). This suggests that *laeA* expression is not solely responsible for the altered expression of secondary metabolite genes, particularly in *A. fumigatus* ∆*gliT* when it is exposed to exogenous gliotoxin. Expression of *A. fumigatus gliT* in ∆*laeA* [32] exposed to exogenous gliotoxin for 3 h was analysed. *A. fumigatus gliT* expression increased following exogenous gliotoxin exposure in ∆*laeA* (Figure 8) indicating that loss of *laeA* does not affect *gliT* expression in the presence of exogenous gliotoxin. Recently, the global regulatory *velvet* gene, *A. fumigatus veA*, has been implicated in secondary metabolite biosynthesis regulation, particularly with respect to gliotoxin [20] and fumagillin [22]. However, from the data presented here, *A. fumigatus veA* is not differentially regulated in either *A. fumigatus* wild-type or *A. fumigatus* ∆*gliT* in exogenous gliotoxin presence. *A. fumigatus gliT* expression in *A. fumigatus* ∆*veA* [34] was assessed following exposure to exogenous gliotoxin and was increased in ∆*veA* in the presence of exogenous gliotoxin (Figure 8). *A. fumigatus gliT* expression was higher in the methanol control (Figure 8) compared to the other mutants under the same conditions. Expression of *A. fumigatus gliT* in ∆*veA* grown in media only was lower than the methanol control (data not shown), suggesting that methanol induced increased *gliT* expression in *A. fumigatus* ∆*veA*. The increased *gliT* expression in *A. fumigatus* ∆*veA* following methanol addition was only observed in this strain, and was not observed in other deletion strains generated in this background (e.g. ∆*laeA*).

The expression of *A. fumigatus gliT* was also investigated in deletion mutants with abrogated fumitremorgin B, fumagillin and pseurotin A production namely, *A. fumigatus* ∆*fapR*, ∆*fmaA* and *∆psoA*, respectively [27]. As was the case in *A. fumigatus laeA*, expression of *A. fumigatus gliT* increased in these mutants following exogenous gliotoxin exposure (Figure 8) indicating that loss of production of these metabolites does not affect *gliT* expression.

### Siderophore-iron transport and siderophore biosynthesis is affected by exogenous gliotoxin and to a greater extent in *A. fumigatus*∆*gliT*

Functional classification of the differentially regulated gene set revealed an enrichment of genes involved in siderophore-iron transport in both *A. fumigatus* wild-type and *A. fumigatus* ∆*gliT* when exposed to exogenous gliotoxin. In *A. fumigatus* wild-type exposed to exogenous gliotoxin, expression of six siderophore-iron transport genes was up-regulated, while in *A. fumigatus ∆gliT,* expression of two siderophore-iron transport genes was up-regulated and 12 genes are down-regulated (Table 3). *A. fumigatus sidF* was *de novo* expressed in *A. fumigatus* wild-type upon exposure to exogenous gliotoxin. Expression of *A. fumigatus mirD* was up-regulated in both *A. fumigatus* wild-type (log_2_ 2.57-fold) and *A. fumigatus* ∆*gliT* (log_2_ 3.07-fold) in exogenous gliotoxin presence. Interestingly, three genes that were significantly up-regulated in *A. fumigatus* wild-type exposed to exogenous gliotoxin were significantly down-regulated in *A. fumigatus* ∆*gliT*, when challenged with exogenous gliotoxin. *A. fumigatus fre7* was significantly up-regulated log_2_ 2.80-fold in *A. fumigatus* wild-type, but significantly down-regulated log_2_ 2.77-fold in *A. fumigatus* ∆*gliT*. AFUA_3G13670 and AFUA_3G13700, significantly up-regulated log_2_ 7.03- and log_2_ 7.62-fold respectively, in *A. fumigatus* wild-type in exogenous gliotoxin presence, were significantly down-regulated upon exogenous gliotoxin exposure log_2_ 7.06- and log_2_ 7.82-fold in *A. fumigatus* ∆*gliT*. Expression of AFUA_3G13700 in both *A. fumigatus* wild-type and ∆*gliT* exposed to exogenous gliotoxin, was confirmed by qRT-PCR (Figure 5).

**Table 3:** **Log**_**2**_ **(fold change) in siderophore-iron transport gene and siderophore biosynthetic gene expression in** ***A. fumigatus*** **wild-type and ∆** ***gliT*** **exposed to exogenous gliotoxin**

		Wild-type_Glio v Wild-type_MeOH		∆***gliT***_Glio v ∆***gliT***_MeOH	
Gene	Gene name	Log_2_(fold change)	q value	Log_2_(fold change)	q value
**Siderophore biosynthesis genes**					
AFUA_1G17190	*sidI*	2.393	1.000	0.859	1.000
AFUA_1G04450	*sidL*	−0.156	0.969	−0.148	0.798
AFUA_1G17200	*sidC*	1.247	0.105	−0.232	0.694
AFUA_2G07680	*sidA*	1.066	0.155	2.157	0.0005
AFUA_2G08590	*pptA*	0.534	0.738	0.854	0.143
AFUA_3G03400	*sidF*	Present^a^	0.005	4.968	0.227
AFUA_3G03410	*sidH*	3.013	0.172	Present^b^	0.0005
AFUA_3G03420	*sidD*	4.657	1.000	2.815	1.000
AFUA_3G03650	*sidG*	Present^a^	1.000	Present^b^	1.000
AFUA_3G03660	*estB*	1.113	1.000	5.852	0.308
AFUA_5G11260	*sreA*	−0.740	0.510	−1.152	0.014
**Siderophore-iron transport genes**					
AFUA_1G01430	-	−1.371	0.080	−2.561	0.0005
AFUA_1G14340	-	0.206	0.957	−3.197	0.0005
AFUA_1G16040	-	0.081	0.990	−2.115	0.004
AFUA_2G01270	-	0.011	0.997	−1.605	0.031
AFUA_3G01360	-	0.251	0.969	−2.778	0.045
AFUA_3G02670	-	−1.506	0.054	−2.514	0.0005
AFUA_3G03400	*sidF*	Present^a^	0.005	4.968	0.227
AFUA_3G03440	*mirD*	2.569	0.005	3.075	0.0005
AFUA_3G13670	*-*	7.033	0.005	−7.058	0.0005
AFUA_3G13700	*-*	7.619	0.005	−7.824	0.0005
AFUA_4G03940	*fre7*	2.803	0.005	−2.765	0.005
AFUA_6G02170	-	−1.026	0.189	−3.820	0.0005
AFUA_6G02820	-	0.290	0.926	−2.396	0.0005
AFUA_6G13750	-	2.256	0.005	−0.998	0.146
AFUA_7G06060	*sit1*	0.927	0.306	2.785	0.0005
AFUA_8G06210	-	Present^a^	1.000	Absent^c^	0.0005

^a^ Switched on in ATCC46645_Glio, ^b^ switched on in ∆*gliT*_Glio, ^c^Gene expression absent in ∆*gliT*_Glio.

The observed differential regulation of genes involved in siderophore-iron transport prompted us to review whether expression of siderophore biosynthetic genes [35] was affected by exogenous gliotoxin in *A. fumigatus* wild-type and *A. fumigatus* ∆*gliT*. Indeed we have already noted that *sidF* expression, a transacylase that transfers anhydromevalonyl to hydroxyornithine during extracellular siderophore biosynthesis [36], was activated in *A. fumigatus* wild-type following exogenous gliotoxin exposure. In *A. fumigatus* ∆*gliT*, expression of two genes involved in siderophore biosynthesis was differentially regulated in response to exogenous gliotoxin (Table 3). *A. fumigatus sidH*, involved in providing the anhydromevalonyl-CoA moiety for extracellular siderophore biosynthesis [37], was *de novo* expressed while *A. fumigatus sidA*, which is required for the first step of siderophore biosynthesis [38], was significantly up-regulated log_2_ 2.16-fold (*p* =0.0005). qRT-PCR analysis of the expression of *A. fumigatus sidH* confirmed the increased expression in *A. fumigatus* ∆*gliT* following exogenous gliotoxin exposure (Figure 5).

### Nitrogen metabolism is down-regulated in *A. fumigatus*∆*gliT*in response to exogenous gliotoxin

In *A. fumigatus* ∆*gliT* exposed to exogenous gliotoxin, expression of 55 genes involved in nitrogen metabolism was down-regulated, while expression of 4 genes was up-regulated (Table 4). In contrast, 3 genes were up-regulated in *A. fumigatus* wild-type and 8 genes were down-regulated in response to exogenous gliotoxin (Table 4). In *A. fumigatus* ∆*gliT*, expression of two genes, *cyp5081B1*, a putative cytochrome P450 monooxygenase already mentioned in helvolic acid biosynthesis [25], and AFUA_6G00412, predicted to have amino acid transmembrane transporter activity [39] was completely abrogated in response to exogenous gliotoxin. Expression of *A. fumigatus optB* and *cpsI*, which are induced when BSA is the sole nitrogen source [40], was down-regulated log_2_ 5.399- and log_2_ 3.98-fold, respectively, in *A. fumigatus* ∆*gliT* exposed to exogenous gliotoxin. The decreased expression of *A. fumigatus optB* in *A. fumigatus* ∆*gliT* exposed to exogenous gliotoxin was confirmed by qRT-PCR (Figure 5). When utilising complex nitrogen sources, *A. fumigatus* secretes proteases, and increases expression of amidase, aminotransferase, and amino acid and peptide transporter genes [40, 41]. These secreted proteases are regulated by a conserved regulatory factor, *prtT* [40, 41], which is decreased log_2_ 3.66-fold in expression in ∆*gliT* but unchanged in wild-type following exogenous gliotoxin exposure. Indeed, further examination revealed decreased expression of 14 proteases, 3 amidases and 7 amino acid and peptide transporters in ∆*gliT* exposed to exogenous gliotoxin, while expression of one amidase, AFUA_5G09140, was increased log_2_ 3.37-fold (Table 4). Expression of both nitrate transporters, *crnA* and *nrtB*, which are predicted gene pairs [42], was significantly down-regulated (log_2_ 2.689- and log_2_ 4.504-fold) in *A. fumigatus* ∆*gliT* upon exogenous gliotoxin exposure. Expression of a number of genes with predicted involvement in the oxidation-reduction process was down-regulated in *A. fumigatus* ∆*gliT* only following exogenous gliotoxin exposure. Among these genes were nitronate monooxygenases (AFUA_2G17430 and AFUA_4G07940 down-regulated log_2_ 4.444- and log_2_ 2.304-fold) and copper ion binding domain genes (AFUA_1G13440, AFUA_5G01470 and AFUA_5G07360; down-regulated log_2_1.692-, log_2_ 4.125- and log_2_ 3.948-fold, respectively). In *A. fumigatus* wild-type exposed to exogenous gliotoxin, two predicted carbon-nitrogen ligases, AFUA_4G04160 and AFUA_4G04170, were down-regulated log_2_ 1.926- and log_2_ 2.622-fold, respectively. These carbon-nitrogen ligases are not differentially regulated in *A. fumigatus* ∆*gliT* upon exogenous gliotoxin exposure, suggesting that loss of *gliT* may hinder the down-regulation of these genes in response to gliotoxin.

**Table 4:** **Log**_**2**_ **(fold change) in nitrogen metabolic gene, protease gene, amidase gene and animo acid transporter gene expression in** ***A. fumigatus*** **wild-type and ∆** ***gliT*** **exposed to exogenous gliotoxin**

		Wild-type_Glio v Wild-type_MeOH		∆gliT_Glio v ∆gliT_MeOH	
Gene	Gene name	Log_2_(fold_change)	q_value	Log_2_(fold_change)	q_value
AFUA_1G02780	*-*	−2.005	0.064	−1.839	0.004
AFUA_1G04160	*-*	−1.796	0.015	−3.834	0.0005
AFUA_1G04560	*-*	−0.364	0.922	−2.161	0.0005
AFUA_1G11250	*-*	−0.610	0.695	−3.225	0.0005
AFUA_1G12850	*crnA*	0.276	0.963	−2.689	0.002
AFUA_1G13220	*-*	0.455	0.835	−3.209	0.0005
AFUA_1G17470	*nrtB*	−0.098	0.990	−4.504	0.013
AFUA_2G02020	*-*	−0.366	0.962	−4.403	0.0005
AFUA_2G02250	*-*	−0.518	0.895	−2.276	0.007
AFUA_2G03900	*-*	−0.355	0.910	−3.894	0.0005
AFUA_2G10520	*uaZ*	−0.780	0.834	−2.793	0.002
AFUA_2G10560	*-*	−0.135	0.988	−4.450	0.0005
AFUA_2G12900	*-*	−0.822	0.556	−1.577	0.003
AFUA_2G15240	*optB*	0.002	0.999	−5.399	0.0005
AFUA_2G17430	*-*	−0.680	0.736	−4.444	0.005
AFUA_3G07040	*cps1*	−0.120	0.984	−3.980	0.0005
AFUA_4G01230	*-*	−0.149	0.973	−4.195	0.0005
AFUA_4G03770	*-*	−1.008	0.311	−2.329	0.0005
AFUA_4G04160	*-*	−1.926	0.033	−0.593	0.319
AFUA_4G04170	*-*	−2.622	0.005	−0.820	0.144
AFUA_4G07940	*-*	−0.943	0.527	−2.304	0.008
AFUA_4G14790	*cyp5081B1*	1.993	0.228	Absent^a^	0.0005
AFUA_5G00710	*-*	0.605	0.691	−3.021	0.0005
AFUA_5G01360	*-*	−0.498	0.891	−3.701	0.024
AFUA_5G06230	*-*	−1.355	0.503	−4.916	0.0005
AFUA_5G07520	*-*	−0.378	0.922	−2.654	0.014
AFUA_5G13810	*-*	−0.423	0.895	−1.715	0.029
AFUA_5G15050	*-*	−1.577	0.362	−3.783	0.010
AFUA_6G00412	*-*	0.915	1.000	Absent^a^	0.0005
AFUA_6G01920	*-*	−1.011	0.433	−1.887	0.004
AFUA_6G02030	*-*	−0.313	0.926	−4.194	0.0005
AFUA_6G02210	*-*	−1.136	0.161	−3.475	0.0005
AFUA_6G05020	*-*	−0.816	0.339	−1.510	0.002
AFUA_6G08000	*gmdA*	−1.332	0.293	−2.252	0.0005
AFUA_6G10210	*-*	−0.664	0.708	−2.153	0.0005
AFUA_7G00910	*optH*	−0.132	0.986	−2.089	0.007
AFUA_7G01690	*-*	−1.322	0.269	−1.753	0.0005
AFUA_7G02070	*nfr1*	−0.453	0.839	−3.250	0.0005
AFUA_7G03850	*-*	−1.776	0.018	−3.643	0.0005
AFUA_8G00190	*ftmC*	−1.278	0.205	−5.681	0.0005
AFUA_8G01570	*-*	−1.158	0.561	−3.979	0.003
AFUA_8G01780	*-*	−1.171	0.639	−2.758	0.036
AFUA_8G04340	*-*	−0.308	0.910	−1.530	0.001
AFUA_8G05220	*-*	0.048	0.996	−2.941	0.0005
AFUA_8G06580	*-*	0.118	0.988	−4.540	0.0005
AFUA_1G13440	*-*	0.520	0.792	−1.692	0.010
AFUA_3G00680	*-*	−4.122	0.005	−1.910	0.0005
AFUA_3G14590	*-*	−2.555	0.005	−2.518	0.0005
AFUA_4G00630	*-*	−1.078	0.246	−3.158	0.0005
AFUA_4G09840	*-*	−1.815	0.028	−3.253	0.0005
AFUA_5G01470	*-*	−0.769	0.739	−4.125	0.001
AFUA_5G07360	*-*	−1.768	0.138	−3.948	0.0005
AFUA_7G04180	*-*	−3.796	0.005	−0.913	0.156
AFUA_8G01580	*-*	−1.324	0.074	−5.553	0.0005
AFUA_8G06470	*-*	−0.077	0.990	−4.663	0.0005
AFUA_5G01450	*-*	−1.259	0.122	−5.973	0.0005
AFUA_5G08910	*-*	−1.162	0.102	−3.491	0.0005
AFUA_7G06270	*-*	−1.038	0.309	−2.581	0.0005
AFUA_1G10820	*-*	0.846	0.311	1.730	0.0005
AFUA_2G15590	*-*	1.173	0.130	2.559	0.0005
AFUA_3G06540	*met16*	−0.276	0.942	3.029	0.0005
AFUA_6G08920	*-*	−0.570	0.716	2.019	0.0005
AFUA_4G01440	*-*	2.655	0.018	−1.625	0.251
AFUA_5G01662	*-*	2.626	0.005	−0.025	0.974
AFUA_7G05500	*gstB*	1.991	0.005	−1.228	0.021
AFUA_2G09030	*dppV*	0.411	0.814	−4.442	0.0005
AFUA_4G09320	*dppIV*	1.202	0.347	−6.695	0.0005
AFUA_3G00650	*lap2*	−0.065	0.990	−1.608	0.008
AFUA_2G07500	*pepP*	−0.790	0.447	−1.933	0.0005
AFUA_4G03490	*-*	−0.845	0.649	−2.546	0.0005
AFUA_2G17330	*-*	−0.543	0.707	−1.719	0.001
AFUA_2G01250	*-*	−0.683	0.745	−1.989	0.0005
AFUA_6G13540	*cp3*	−0.474	0.839	−1.549	0.008
AFUA_6G00310	*cpdS*	−0.748	0.531	−3.976	0.0005
AFUA_5G13300	*pep1*	−0.833	0.678	−4.357	0.0005
AFUA_3G02970	*gprA*	−0.228	0.979	−5.119	0.0005
AFUA_5G01200	*cp6*	−0.621	0.834	−4.932	0.0005
AFUA_8G00410	*metAP*	−0.113	0.987	−5.196	0.0005
AFUA_5G09140	*-*	−0.443	0.910	3.372	0.002
AFUA_5G02990	*-*	−0.805	0.512	−4.047	0.0005
AFUA_1G09120	*-*	−0.272	0.962	−2.985	0.002
AFUA_8G00800	*-*	0.641	0.899	−6.795	0.0005
AFUA_1G12240	*-*	0.347	0.964	−5.161	0.0005
AFUA_8G02550	*-*	0.564	0.819	−8.305	0.003
AFUA_7G04290	*-*	−0.464	0.910	−2.355	0.0005
AFUA_8G05860	*-*	−0.463	0.861	−2.099	0.003

^a^Gene expression absent in ∆*gliT*_Glio.

### Exogenous gliotoxin exposure alters gene expression involved in ribosome biogenesis and translation in *A. fumigatus*∆*gliT*while transcription related genes are down-regulated

Dysregulation of ribosome biogenesis and translation was observed in *A. fumigatus* ∆*gliT* following exogenous gliotoxin exposure, whereby expression of 20 genes was up-regulated and two genes down-regulated (Table 5). Of the 20 genes up-regulated in *A. fumigatus* ∆*gliT* in response to exogenous gliotoxin, 7 genes encode 60S ribosomal proteins and two encode 40S ribosomal proteins, all of which are increased log_2_1.5-1.7-fold in expression (Table 5). The two genes down-regulated in response to exogenous gliotoxin in *A. fumigatus* ∆*gliT* have already been mentioned as secondary metabolite biosynthetic genes. *A. fumigatus metAP* in the fumagillin biosynthetic gene cluster [30] was down-regulated log_2_ 4.340-fold and *A. fumigatus elfB* in the pseurotin A biosynthetic gene cluster was down-regulated log_2_ 7.265-fold [27, 31]. In contrast to *A. fumigatus* ∆*gliT*, only two genes involved in ribosome biogenesis and translation were up-regulated in *A. fumigatus* wild-type exposed to exogenous gliotoxin (Table 5). AFUA_5G07340 was up-regulated log_2_ 1.909-fold while AFUA_2G12150 was up-regulated log_2_ 2.417-fold. In addition to the dysregulation of ribosome biogenesis and translation, 44 genes involved in transcription were also down-regulated in *A. fumigatus* ∆*gliT* in response to exogenous gliotoxin, compared to 3 down-regulated genes in wild-type exposed to exogenous gliotoxin (Table 6). A proteomic investigation of *A. fumigatus* ∆*gliT*^*ATCC26933*^ reflected this alteration in translation whereby 6 proteins were uniquely expressed or increased in abundance and 15 proteins were absent or decreased in abundance when exposed to exogenous gliotoxin (Tables 7 and 8). Loss of GliT negatively impacts expression of genes involved in transcription processes and results in significantly decreased transcription of genes in the presence of exogenous gliotoxin, culminating in disruption of translation.

**Table 5:** **Log**_**2**_ **(fold change) in ribosome biogenesis and translation gene expression in** ***A. fumigatus*** **wild-type and ∆** ***gliT*** **exposed to exogenous gliotoxin**

		Wild-type_Glio v Wild-type_MeOH		∆gliT_Glio v ∆gliT_MeOH	
Gene	Gene name	Log_2_(fold_change)	q_value	Log_2_(fold_change)	q_value
AFUA_3G05600	*-*	1.122	0.228	1.647	0.004
AFUA_3G06760	*-*	1.249	0.075	1.763	0.001
AFUA_5G07340	*-*	1.909	0.012	0.268	0.728
AFUA_6G05200	*-*	1.061	0.227	1.590	0.008
AFUA_6G13250	*-*	1.203	0.117	1.609	0.003
AFUA_8G00410	*metAP*	−0.113	0.987	−4.340	0.001
AFUA_8G00580	*elfB*	0.306	0.964	−7.265	0.003
AFUA_2G09200	*-*	1.100	0.143	1.510	0.003
AFUA_3G07360	*-*	0.492	0.739	1.506	0.004
AFUA_1G12890	*-*	1.191	0.212	1.595	0.006
AFUA_1G15020	*-*	1.036	0.277	1.545	0.008
AFUA_2G10300	*-*	1.353	0.060	1.537	0.004
AFUA_2G12150	*-*	2.417	0.005	0.561	0.422
AFUA_3G08080	*-*	0.214	0.952	1.655	0.001
AFUA_4G07250	*-*	0.599	0.589	1.572	0.002
AFUA_4G07730	*-*	1.190	0.163	1.592	0.004
AFUA_4G11990	*-*	0.309	0.891	1.682	0.001
AFUA_5G05630	*-*	1.222	0.122	1.661	0.002
AFUA_5G06430	*-*	0.559	0.649	1.501	0.002
AFUA_6G02440	*-*	1.006	0.272	1.658	0.002
AFUA_6G02450	*-*	0.714	0.493	1.564	0.003
AFUA_6G11260	*-*	1.105	0.232	1.532	0.008
AFUA_6G12960	*-*	0.521	0.686	1.721	0.001
AFUA_2G10100	*aspf8*	1.295	0.138	1.580	0.007

**Table 6:** **Log**_**2**_ **(fold change) in transcription gene expression in** ***A. fumigatus*** **wild-type and ∆** ***gliT*** **exposed to exogenous gliotoxin**

		Wild-type_Glio v Wild-type_MeOH		∆gliT_Glio v ∆gliT_MeOH	
Gene	Gene name	Log_2_(fold_change)	q_value	Log_2_(fold_change)	q_value
AFUA_4G11480	*-*	−0.766	0.446	−1.532	0.003
AFUA_6G05160	*azf1*	−1.541	0.043	−0.251	0.753
AFUA_6G12150	*-*	0.502	0.913	−6.233	0.001
AFUA_7G01340	*-*	0.082	0.990	−2.982	0.001
AFUA_4G02930	*-*	0.450	0.796	−1.971	0.001
AFUA_3G10120	*-*	−0.819	0.488	−1.669	0.003
AFUA_4G10110	*htfA*	0.105	0.986	−1.565	0.012
AFUA_1G10080	*zafA*	−0.434	0.843	−1.535	0.001
AFUA_1G01240	*-*	−0.561	0.770	−1.702	0.025
AFUA_1G14945	*-*	−0.839	0.505	−1.754	0.026
AFUA_2G04262	*-*	−1.644	0.012	−0.759	0.171
AFUA_2G11180	*flbA*	−0.126	0.977	−1.511	0.003
AFUA_3G03330	*-*	−2.132	0.021	−0.739	0.221
AFUA_5G03780	*-*	1.277	0.320	−2.864	0.001
AFUA_5G09720	*-*	−1.780	0.086	−1.586	0.033
AFUA_5G14530	*-*	0.120	0.987	−2.166	0.010
AFUA_8G01150	*-*	0.037	0.997	−3.345	0.001
AFUA_8G01940	*-*	−1.248	0.221	−1.846	0.027
AFUA_8G02720	*-*	−0.032	0.997	−1.848	0.001
AFUA_8G04130	*farB1*	−0.496	0.798	−1.898	0.001
AFUA_8G06460	*-*	−0.005	0.998	−1.741	0.009
AFUA_6G11740	*-*	−0.919	0.475	−7.145	0.004
AFUA_2G05180	*-*	−0.361	0.891	−3.819	0.001
AFUA_1G16600	*-*	−0.343	0.964	−4.481	0.001
AFUA_8G00280	*-*	−0.720	0.574	−1.813	0.007

**Table 7:** **Proteins with increased abundance in, or unique to,** ***A. fumigatus*** **∆** ***gliT*** ^**ATCC26933**^ **with gliotoxin compared to the methanol control**

Protein IDs	Log_2_(fold increase)	***p***value	Peptides	Sequence coverage [%]
AFUA_6G06470	Unique	N/A	6	58.5
AFUA_1G09510	Unique	N/A	2	34.5
AFUA_4G03140	Unique	N/A	4	12.5
AFUA_3G14540	2.279	1.52E-02	6	38.3
AFUA_1G15270	1.135	2.05E-02	33	43.1
AFUA_7G00350*	1.119	1.49E-02	6	23.2

*Gene expression also significantly up-regulated in RNA-seq analysis.

**Table 8:** **Proteins with decreased abundance in, or absent from, ∆** ***gliT*** ^**ATCC26933**^ **with gliotoxin compared to the methanol control**

Protein IDs	Log_2_(fold decrease)	***p***value	Peptides	Sequence coverage [%]
AFUA_3G00330*	Unique	N/A	6	36.2
AFUA_5G14000*	Unique	N/A	7	61.3
AFUA_1G10960	Unique	N/A	2	18.2
AFUA_8G00540*	3.032	1.72E-02	37	18.7
AFUA_2G15290	2.788	6.36E-04	6	81.6
AFUA_8G00440*	2.461	7.89E-04	12	21.2
AFUA_7G06420	2.451	3.18E-03	18	63.5
AFUA_8G00550*	2.016	1.03E-02	12	63.8
AFUA_2G04060*	1.799	5.53E-03	12	58.7
AFUA_3G03350*	1.603	2.31E-03	50	38.6
AFUA_5G07170*	1.361	3.93E-02	7	44.9
AFUB_044910	1.234	1.02E-02	16	61.3
AFUA_1G01010	1.128	4.74E-02	33	26.4
AFUA_8G05580	1.091	1.12E-02	11	43.2
AFUA_6G10120	1.072	2.60E-02	9	46.4

*Gene expression also significantly down-regulated in RNA-seq analysis.

## Discussion

To dissect the role played by the gliotoxin oxidoreductase, *gliT*, in self-protection against gliotoxin, a comparative transcriptomic analysis of the impact of exogenous gliotoxin on *A. fumigatus* wild-type and ∆*gliT* was carried out via RNA-seq analysis. To reduce the affects of endogenous gliotoxin, a low gliotoxin-producing background strain, *A. fumigatus* ATCC46645 [2] was employed, along with Sabouraud-Dextrose media which is non-permissive for gliotoxin biosynthesis [5]. Exogenous gliotoxin exposure resulted in changed expression of 164 genes in *A. fumigatus* wild-type. However, altered expression of over 1700 genes was observed in *A. fumigatus* ∆*gliT*. Closer inspection revealed alterations in expression of clusters encoding secondary metabolites, particularly gliotoxin, helvolic acid, fumitremorgin, fumagillin and pseurotin A biosynthesis genes, siderophore biosynthesis genes, ribosome biogenesis genes and genes involved in translation and nitrogen metabolism.

Exogenous gliotoxin induces the expression of gliotoxin biosynthetic genes in both *A. fumigatus* wild-type and ∆*gliT*. Expression of *gliZ* was increased in expression in wild-type, along with *gliP*, the bimodular non-ribosomal peptide synthetase that produces the cyclo-L-Phe-L-Ser diketopiperazine intermediate [9, 10, 43]. Up-regulation of *gliZ* and *gliP* expression in particular, along with *gliF* suggests that exogenous gliotoxin induces *de novo* gliotoxin production in wild-type. Indeed this has been shown to be the case in *A. fumigatus* Af293 where addition of exogenous gliotoxin under gliotoxin-inducing culture conditions resulted in detection of *de novo* gliotoxin production determined by the use of [^13^C]-Phenylalanine and LC-MS analysis. Recently, cyclo-L-Phe-L-Ser was determined to be a major component of the metabolome and was detected in lung tissue of infected mice [11, 44]. The production of this compound may be increased consequent to exogenous gliotoxin exposure in wild-type due to up-regulation of *gliP* expression. It has been demonstrated that *A. fumigatus gliT* expression is not solely under the control of *gliZ* [2], and so up-regulation of *gliZ* expression is not absolutely required to induce *gliT* expression for the purpose of self-protection. While *gliZ* expression is not significantly altered in ∆*gliT* following exogenous gliotoxin exposure in the RNA-seq analysis, qRT-PCR demonstrated increased expression under these conditions. Expression of a number of *gli* genes was up-regulated in ∆*gliT* following exogenous gliotoxin exposure, including *gliM*, which was not altered in wild-type. *A. fumigatus gliM* is a predicted *O*-methyltransferase [3] and methylation has been proposed as a method of self-protection against disulfide bridge-containing metabolites. Holomycin contains a disulfide bridge similar to gliotoxin [16, 17, 45] and *S*-methylation has been proposed as an alternative method of self-protection against this antibiotic and its biosynthetic intermediates in *S. clavuligerus* upon deletion of the dithiol oxidase, *HlmI*, which is functionally homologous to *A. fumigatus gliT* [17, 45]. In *Y. ruckeri*, an RNA methyltransferase, *Hom12*, methylates RNA in a proposed self-protection mechanism against the cytotoxic effects of holomycin [16]. Interestingly, in *A. fumigatus* ∆*gliT* following exogenous gliotoxin exposure, a tRNA methyltransferase, AFUA_4G12280, is up-regulated log_2_ 2.56-fold, while its expression is unaltered in wild-type in the presence of exogenous gliotoxin. The increased expression of these methyltransferases, among others, in *A. fumigatus* ∆*gliT* following exposure to exogenous gliotoxin may suggest possible alternative functionalities, or self-protection mechanisms against gliotoxin in the absence of GliT. Interestingly, a newly-identified methyltransferase, gliotoxin methyltransferase A (GtmA), has been demonstrated to play a role in attenuating gliotoxin biosynthesis [46]. GtmA (AFUA_2G11120) expression is significantly up-regulated (*p* <0.00005) by gliotoxin exposure (Additional file 1).

In addition to up-regulation of the *gli* cluster, dysregulation of other secondary metabolite gene clusters was observed upon exogenous gliotoxin addition, particularly to *A. fumigatus* ∆*gliT*. Up-regulated expression of two genes; *osc3* and *sdrI*, from the gene cluster encoding the fusidane antibiotic helvolic acid was observed upon exogenous gliotoxin addition to *A. fumigatus* wild-type [25]. However, exogenous gliotoxin addition to *A. fumigatus* ∆*gliT* resulted in down-regulated expression of two genes and abrogation of the expression of *cyp5081D1*, suggesting that *gliT* deletion, along with exogenous gliotoxin exposure negatively regulates the helvolic acid gene cluster. In an *A. fumigatus gliZ* complemented strain, helvolic acid production was detectable at 37°C, whereas no helvolic acid was detectable in wild-type or ∆*gliZ* strains at an identical growth temperature [10], suggesting that *gliZ* may be involved in regulating helvolic acid production. Indeed in the present study in wild-type, *gliZ* expression was up-regulated upon gliotoxin exposure and the helvolic acid biosynthetic genes, including *osc3* which encodes the protein that catalyses the first biosynthetic step of helvolic acid biosynthesis [25], are also up-regulated, whereas in ∆*gliT*, the helvolic acid biosynthesis genes are down-regulated. However, we cannot unambiguously assign *gliZ* functionality to hevolic acid production because consistently discrepant *gliZ* expression data was obtained by RNA-seq and qRT-PCR (Figure 5).

The combined loss of *gliT* and exposure to exogenous gliotoxin resulted in attenuated expression of the biosynthetic genes for the secondary metabolites: fumitremorgin B, fumagillin, and pseurotin A. Secondary metabolite production in fungi is generally dependant on the presence of a key backbone enzyme, namely a PKS or a NRPS [47]. In *A. fumigatus* ∆*gliT* exposed to exogenous gliotoxin, the NRPS *ftmA* in the fumitremorgin B cluster, the PKS, *fma-PKS* in the fumagillin cluster and the PKS-NRPS hybrid *psoA/nrps14* in the pseurotin A cluster were all significantly down-regulated suggesting production of these secondary metabolites is also decreased. *A. fumigatus ftmA* encodes the enzyme required for the first biosynthetic step of fumitremorgin synthesis, the synthesis of the diketopiperazine, brevianamide F [29], and is significantly down-regulated log_2_ 6.640-fold in *A. fumigatus* ∆*gliT* following exogenous gliotoxin addition. Expression of the cytochrome P450 *A. fumigatus ftmE*, which was absent in ∆*gliT* exposed to exogenous gliotoxin, encodes the enzyme responsible for formation of fumitremorgin C [48], while another cytochrome P450, *ftmG*, which encodes the enzyme that dihydroxylates the fumitremorgin B intermediate was significantly down-regulated log_2_ 6.519-fold [48]. We hypothesise that fumitremorgin B biosynthesis is decreased, if not abrogated, in *A. fumigatus* ∆*gliT* when exposed to exogenous gliotoxin given the decreased expression of genes essential for synthesis. While determination of fumitremorgin B levels was not successful, measurement of fumitremorgin C and related compounds [48] was carried out in *A. fumigatus* wild-type and ∆*gliT* cultured under secondary metabolite-inducing conditions. Significant reductions in the levels of tryprostatin A and tryprostatin B were observed in *A. fumigatus* ∆*gliT* compared to wild-type, while there was no significant difference in levels of fumitremorgin C between the two strains. Therefore, GliT loss disrupts the production of brevianamide F, tryprostatin A and tryprostatin B, from the fumitremorgin biosynthetic pathway in *A. fumigatus*. The PKS, *fma-PKS*, down-regulated log_2_ 7.287-fold in *A. fumigatus* ∆*gliT*, is essential for fumagillin biosynthesis [30]. Additionally, the C6 type transcription factor *fapR/fumR*, which controls the expression of the other fumagillin cluster genes [22], was down-regulated log_2_ 5.826-fold in *A. fumigatus* ∆*gliT* following exogenous gliotoxin addition. Decreased expression of these two genes, in addition to others in the biosynthetic gene cluster, suggests down-regulation of fumagillin biosynthesis as a direct consequence of *A. fumigatus gliT* loss combined with exogenous gliotoxin stress. Indeed supporting the hypothesis of *A. fumigatus gliT* involvement in facilitating fumagillin biosynthesis, under secondary metabolite-inducing conditions we observed a significant decrease in the production of fumagillin in *A. fumigatus* ∆*gliT* compared to wild-type. It has been determined that the biosynthetic genes for fumagillin and pseurotin A are physically intertwined [27] and these authors also revealed that *fapR*, which controls expression of the fumagillin biosynthesis genes, also controls the expression of pseurotin A biosynthesis genes [27]. Indeed, with the exception of one gene, expression of the pseurotin A biosynthetic cluster was significantly down-regulated in *A. fumigatus* ∆*gliT* upon exogenous gliotoxin addition. *psoA/nrps14* is essential for pseurotin A biosynthesis, as demonstrated by Maiya *et al.* [28], while over-expression of this gene increased pseurotin A accumulation. In *A. fumigatus* ∆*gliT* exposed to exogenous gliotoxin, *psoA/nrps14* expression was down-regulated log_2_ 5.826-fold, while in the proteomic investigation*,* PsoA/nrps14 was decreased log_2_ 3.032-fold in abundance. In addition to PsoA/nrps14, two other proteins required for pseurotin A biosynthesis [27] were decreased in abundance upon exogenous gliotoxin exposure in *A. fumigatus* ∆*gliT*. PsoF (AFUA_8G00440) and PsoC (AFUA_8G00550) were decreased log_2_ 2.461- and log_2_ 2.016-fold providing further support to the hypothesis that pseurotin A biosynthesis is down-regulated in the absence of *A. fumigatus gliT* when challenged with exogenous gliotoxin. As was the case with fumagillin, pseurotin A levels were significantly decreased in *A. fumigatus* ∆*gliT* compared to wild-type when cultured under secondary metabolite-inducing conditions again suggesting possible GliT involvement in enabling pseurotin A biosynthesis.

The biosynthetic gene clusters of fumitremorgin, fumagillin and pseurotin are under the control of the global regulator, *A. fumigatus laeA* [26, 27]. *A. fumigatus laeA* is a methyltransferase and regulates chromatin remodelling through this methyltransferase activity [26, 32, 49]. In *A. fumigatus* ∆*gliT* treated with exogenous gliotoxin, *laeA* expression was not significantly altered, suggesting that it is not responsible for the down-regulation of these gene clusters. In *A. nidulans*, LaeA forms a trimeric complex with two members of the velvet protein family; VeA and VelB, and this complex up-regulates secondary metabolism and sexual development [50]. The complex was subsequently identified and characterised in *A. fumigatus* [34]. *A. fumigatus veA* has been demonstrated to regulate fumagillin and gliotoxin production [20, 22], however *veA* expression was not significantly altered in *A. fumigatus* ∆*gliT* following exogenous gliotoxin production. The bZip transcriptional enhancer RsmA has been shown to positively regulate gliotoxin biosynthesis [44], however the expression of *A. fumigatus rsmA* was not altered significantly in *A. fumigatus* ∆*gliT*, in the absence or presence of exogenous gliotoxin. Overall, our findings lead us to postulate that the gliotoxin biosynthetic/self-protection capacity (i.e., GliT functionality) is necessary for optimal biosynthesis of selected secondary metabolites in *A. fumigatus*.

Altered expression of siderophore-iron transport and siderophore biosynthetic genes in both *A. fumigatus* wild-type and ∆*gliT* following exogenous gliotoxin exposure suggests a disruption in iron homeostasis or iron sensing. Iron is an essential nutrient that is required for many cellular processes, including as a cofactor for numerous enzymes [35]. In both wild-type and ∆*gliT*, siderophore biosynthesis gene expression was up-regulated in response to exogenous gliotoxin indicating that consequent to exogenous gliotoxin exposure, there is an increased requirement for iron despite sufficient iron availability in the culture media, or else a deficit in iron-sensing. *A. fumigatus sidF* expression was activated in wild-type, while in ∆*gliT*, *sidH* expression was activated and that of *sidA* was up-regulated [35]. Interestingly, up-regulation of *sidA* expression has also been observed in ∆*metR*, an *A. fumigatus* mutant deficient in the transcription factor that regulates sulfur assimilation, under iron sufficient but sulfur deficient conditions [21]. Regulatory cross-talk between secondary metabolism and iron requirement has been reported whereby in a *laeA* mutant deficient in gliotoxin production, decreased expression of a number of the siderophore biosynthesis genes was observed under high iron conditions [26]. These authors concluded that *laeA* was also involved in regulating expression of the siderophore biosynthetic genes and in particular, *sidD*. As discussed, we have observed that the combined effect of exogenous gliotoxin exposure and *gliT* deletion has a significant impact on secondary metabolism, despite *laeA* expression being unaffected, and therefore it is interesting that siderophore biosynthesis is also affected. In *A. fumigatus* wild-type, expression of six siderophore-iron transport genes was up-regulated in response to exogenous gliotoxin, in contrast to up-regulation of two siderophore-iron transport genes in ∆*gliT*. Interestingly, in ∆*gliT*, expression of 12 siderophore-iron transport genes was down-regulated. Taken together, this suggests that deletion of *gliT* in combination with exogenous gliotoxin exposure results in a disruption of, or decrease in, siderophore-iron transport. Amich *et al.* [21] noted up-regulation of siderophore-iron transport genes in ∆*metR* under sulfur-limited but iron replete conditions. Despite observing increased expression of both siderophore biosynthetic genes and siderophore-iron transport genes in ∆*metR*, suggesting iron starvation, the authors noted increased levels of ferricrocin, the intracellular siderophore that is used for transport and storage [38, 51]. Iron is utilised in many processes one of which is iron-sulfur cluster biosynthesis. Iron-sulfur clusters are inorganic cofactors involved in cellular processes including enzyme activity regulation, mitochondrial respiration, ribosome biosynthesis and cofactor biosynthesis [52]. Translocation of iron-sulfur clusters requires glutathione, and depletion has been shown to induce an iron starvation-like response in *Saccharomyces cerevisiae* [53–55]. It has previously been shown that exogenous gliotoxin exposure to both *A. fumigatus* wild-type and ∆*gliT* results in decreased GSH levels [5] and so it is conceivable that this decrease in cellular GSH could impact on iron homeostasis or iron sensing.

*A. fumigatus* encodes two transcription factors, *hapX* and *sreA*, that maintain iron homeostasis whereby *hapX* represses *sreA* expression and subsequently iron-consuming pathways, and activates siderophore biosynthesis during iron-starvation, while *sreA* represses *hapX* during iron-sufficient conditions in a negative feedback loop [35, 56]. Interestingly, induction of *gliT* has been shown before when cultures were shifted from iron-limited to replete conditions in both wild-type and a *sreA* deletion strain [57]. Although expression of *hapX* and *sreA* is unchanged in both wild-type and ∆*gliT* upon exogenous gliotoxin exposure, there is significant interplay between *gliT*, sulfur and iron as demonstrated by the altered expression of siderophore biosynthesis and siderophore-iron transport genes and the decreased GSH levels [5] in *A. fumigatus* ∆*gliT*.

FunCat analysis identified significant enrichment of genes involved in nitrogen metabolism in the down-regulated gene set in ∆*gliT* exposed to exogenous gliotoxin. Fungi can utilise various sources of nitrogen, from easily assimilated sources (e.g., ammonium and glutamate), to more complex secondary sources including amino acids and proteins [40, 58]. Following exposure to exogenous gliotoxin, the expression of *prtT*, the conserved regulator of secreted proteases, was decreased and consequentially, a number of proteases previously identified to be under its control were also decreased in expression, including *alp1* and *pep1* [41]. Additionally, a number of other proteases underwent decreased expression in ∆*gliT* when exposed to exogenous gliotoxin. These proteases, some of which include *dppV*, *dppVI*, *cpdS* and *gprA*, have been shown to be induced when BSA is the sole nitrogen source [41]. In order to conserve energy, fungi will preferentially utilise nitrogen sources that are easily assimilated over complex nitrogen sources [59]. Sabouraud-Dextrose medium contains a pancreatic digest of casein and a peptic digest of animal tissue. As the media already contained digested proteins, we conclude that expression of these secreted proteases is significantly down-regulated in ∆*gliT* exposed to exogenous gliotoxin in order to conserve energy. Interestingly, a number of genes involved in amino acid and peptide transport are also decreased in expression with the exception of one amidase, AFUA_5G09140, the expression of which is up-regulated in ∆*gliT* following exogenous gliotoxin exposure. This was surprising as, despite the availability of amino acids and peptides in the media, the expression of genes encoding enzymes required for uptake of these amino acids and peptides are down-regulated. It is possible that sufficient enzymes are present for adequate uptake of nitrogen sources to compensate for the decreased expression of these genes, or that the down-regulation of these genes is a consequence of the down-regulation of *prtT* and the other secreted proteases suggesting that there is a decreased nitrogen requirement in ∆*gliT*, when exposed to exogenous gliotoxin.

Loss of *A. fumigatus gliT* had a significant impact on the transcriptome when challenged with exogenous gliotoxin whereby expression of 1,700 genes was altered, of which 1192 were down-regulated, that was not observed in wild-type where 164 genes had altered expression. This suggests that consequent to GliT absence, transcription is suppressed in the presence of exogenous gliotoxin. This is further supported by the decreased expression of 44 genes involved in transcription processes, many of which are transcription factors. Further to the effects of *gliT* deletion on transcription, translation is also disrupted in *A. fumigatus* ∆*gliT* following exposure to exogenous gliotoxin. Altered expression of genes required for ribosome biogenesis and translation processes was observed in ∆*gliT*. To support the hypothesis that translation is disrupted, the LFQ proteomic investigation of *A. fumigatus* ∆*gliT* did not reflect the large transcriptome changes, as only 6 proteins with increased abundance or uniquely present, and 15 proteins absent or with decreased abundance were identified. It is important to note that despite the low number of proteins altered in abundance, there is agreement between the RNA-seq analysis and the proteomic analysis of *A. fumigatus* ∆*gliT* exposed to exogenous gliotoxin, whereby the genes encoding one of the proteins increased in abundance and 8 of the proteins decreased in abundance were up-regulated and down-regulated, respectively.

## Conclusions

We present the first global investigation of the transcriptional response to exogenous gliotoxin in *A. fumigatus* wild-type and ∆*gliT* employing RNA-seq analysis. While exogenous gliotoxin elicits some transcriptome remodelling in wild-type, in ∆*gliT*, the transcriptional response is 10-fold that of wild-type, with approximately 70% of these altered genes decreased in expression. We found that the combined loss of *gliT* and exogenous gliotoxin exposure results in decreased expression of a number of secondary metabolite genes from the biosynthetic clusters of helvolic acid, fumitremorgin B, pseurotin A and fumagillin despite the unchanged expression of *laeA*, the global regulator of secondary metabolism. Thus, GliT functionality may extend to enhancing the biosynthesis of selected secondary metabolites in *A. fumigatus*. In addition to this, the decreased expression of many transcription factor genes, along with genes involved in siderophore-iron transport and siderophore biosynthesis and nitrogen metabolism indicates that exogenous gliotoxin induces a starvation-like response despite the use of a rich media. Furthermore, the combined RNA-seq and proteomic analysis revealed deletion of *gliT* abrogated transcription and disrupts translation when exposed to exogenous gliotoxin. Overall, this study provides a detailed overview of the response to exogenous gliotoxin in resistant and sensitive *A. fumigatus* strains, enhances our understanding of the manner in which gliotoxin exerts its affects as a toxin, and provides a unique glimpse into cross-talk between apparently unrelated secondary metabolite gene clusters.

## Methods

### *Aspergillus fumigatus*strain information and growth conditions

Conidia were maintained on malt extract agar plates. *A. fumigatus* wild-type and mutant strains (1 × 10^6^ cfu/ml) were cultured in Sabouraud-Dextrose media at 37°C with shaking at 200 rpm for 21 h in duplicate or triplicate. Gliotoxin (5 μg/ml final) or MeOH (solvent control) was added and the cultures were incubated for a further 3 h. The mycelia were harvested through miracloth, washed with DEPC water and snap frozen in liquid N_2_.

### RNA extraction and mRNA isolation

RNA was isolated from mycelia, ground to a fine powder in liquid N_2,_ using the RNeasy™ Plant Mini Kit (Qiagen), according to the manufacturer’s instructions. RNA integrity was analysed using an Agilent 2100 Bioanalyzer™ and an Agilent RNA 6000 Nano Kit following the manufacturer’s recommendation.

### Library preparation and sequencing

A library was independently prepared for each biological replicate. Two protocols (TruSeq and Illumina mRNA-seq kit) were used for preparing the Illumina transcriptome libraries. For both protocols, polyadenylated mRNA was purified from total RNA using oligo-dT dynabead selection followed by metal ion hydrolysis fragmentation with an RNA fragmentation solution supplied in kits. First strand synthesis, primed using random oligonucleotides, was followed by second strand synthesis with RNaseH and DNA polI to produce double-stranded cDNA using the Illumina mRNA Seq kit or the TruSeq Illumina kit. Template DNA fragments were end-repaired with T4 and Klenow DNA polymerases and blunt-ended with T4 polynucleotide kinase. A single 3′ adenosine was added to the repaired ends using Klenow exo- and dATP to reduce template concatemerization and adapter dimer formation, and to increase the efficiency of adapter ligation. Adapters (containing primer sites for sequencing, and index sequences when using the TruSeq protocol) were then ligated. Libraries made with the TruSeq protocol were amplified by PCR using KAPA HiFi Polymerase (to enrich for properly ligated template strands, to generate enough DNA, and to add primers for flowcell surface annealing). AMPure SPRI beads were used to purify amplified templates before pooling based on quantification using an Agilent Bioanalyser chip. Pooled TruSeq libraries were then pooled and size selected using the Caliper. After adaptor ligation, individual libraries made with the Illumina mRNA-seq kit were size selected using the CaliperLabChip before PCR amplification followed by AMPure SPRI bead clean up and removal of adaptors with a second Caliper run. KAPA Illumina SYBR Fast qPCR kit was used to quantify the Illumina mRNA-seq libraries before pooling. No qPCR was necessary with the TruSeq libraries and instead a final Agilent Bioanalyser chip analysis was run to confirm the dilution of the final pool. The libraries were sequenced on the Illumina HiSeq platform with a read length of 75 bp paired-ended according to manufacturer’s instructions.

### Data processing

The RNA-seq paired-end reads for each biological replicate were aligned independently using Tophat v2.0.4 [23618408] to the *A. fumigatus* Af293 (CADRE 3a) reference genome sequence with default parameters. The numbers of fragments mapped per replicate is given in Additional file 2: Table S13. Differential gene expression analysis was carried out for each sample independently using Cufflinks (cuffdiff) v2.0.2 [20436464] with default parameters against the gene set in Ensembl Genomes release 14 (CADRE genebuild 3a). The sequencing data has been submitted to the European Nucleotide Archive (ENA) under accession ERP001382 (https://www.ebi.ac.uk/ena/data/view/ERP001382&display=html).

### Data analysis

The differentially regulated genes were analysed using FungiFun [60] to establish association with any functions or pathways in comparison with the non-differentially regulated genes. Gene enrichment analysis was carried out on the up-regulated and down-regulated genes, respectively, for FunCat [23] categories and the *A. fumigatus* annotated KEGG pathways [24]. A *p* value cut-off of 0.05 was used.

### Real time PCR (qRT-PCR)

RNA samples were DNase treated using a DNase kit supplied by Sigma-Aldrich. cDNA synthesis was performed using qScript™ cDNASuperMix (Quanta Biosciences) following the kit instructions. Primers used in this study are listed in Additional file 2: Table S14. The constitutively expressed gene, *A. fumigatus calmodulin* (*calm*) [61] was used as a reference gene. qRT-PCR was performed on the LightCycler®2 480 Real-Time PCR System using the LightCycler®1 Sybr Green 1 Master Mix (Roche) as described previously [62]. qRT-PCR reactions for each gene were analysed in triplicate and were carried out for 40 cycles.

### Feeding experiments with [^13^C]-phenylalanine

*A. fumigatus* Af293 was cultured in Czapek-Dox Broth at 37°C with shaking at 200 rpm in duplicate for 24 h before addition of gliotoxin (5 μg/ml final) or methanol (solvent control) and [^13^C]-phenylalanine (10 μg/ml final) or water (solvent control). Cultures were incubated again at 37°C with shaking at 200 rpm. Culture supernatant was removed after 48 h and again after 72 h. Supernatants were chloroform extracted, dried and resolubilised in methanol prior to LC-MS analysis as described previously [2].

### Whole proteome analysis

*A. fumigatus* ∆*gliT*^ATCC26933^ was cultured in Sabouraud-Dextrose media for 21 h followed by gliotoxin (5 μg/ml final) or methanol addition for 3 h (*n* =4 biological replicates for all specimens). Mycelial lysates were prepared in lysis buffer (100 mM Tris–HCl, 50 mM NaCl, 20 mM EDTA, 10% (v/v) Glycerol, 1 mM PMSF, 1 μg/ml pepstatin A, pH 7.5) with grinding, sonication and clarified using centrifugation. The resultant protein lysates were precipitated using TCA/acetone and resuspended in 8 M Urea. After DTT reduction and IAA-mediated alkylation [63], sequencing grade trypsin combined with ProteaseMax surfactant was added. Digested samples were desalted prior to analysis using C_18_ spin columns (Thermo Scientific). All peptide mixtures were analysed via a Thermo Scientific Q-Exactive mass spectrometer coupled to a Dionex RSLCnano. LC gradients ran from 14–35%B (A: 0.1% (v/v) formic acid, B: 80% (v/v) acetonitrile, 0.1% (v/v) formic acid) over 2 h, and data was collected using a Top15 method for MS/MS scans. Comparative proteome abundance and data analysis was performed using MaxQuant software (Version 1.3.0.5) [64], with Andromeda used for database searching and Perseus (Version 1.4.1.3) used to organise the data. Carbamidomethylation of cysteines was set as a fixed modification, while oxidation of methionines and acetylation of N-termini were set as variable modifications. The maximum peptide/protein false discovery rates (FDR) were set to 1% based on comparison to a reverse database. The Label-Free Quantification (LFQ) algorithm was used to generate normalised spectral intensities and infer relative protein abundance. Proteins that matched to a contaminants database or the reverse database were removed and proteins were only retained in final analysis if detected in at least three replicates from at least one sample. Quantitative analysis was performed using a t-test to compare pairs of samples, and proteins with significant change in abundance (*p* value <0.05; fold change ≥2) were included in the quantitative results. Qualitative analysis was also performed, to detect proteins that were found in at least 3 replicates of a particular sample, but undetectable in the comparitor sample.

### Secondary metabolite analysis

*A. fumigatus* wild-type and ∆*gliT*^46645^ were cultured in Czapeks-Dox broth for 96 h at 37°C at 200 rpm in duplicate. Culture supernatants were organically extracted using an equal volume of chloroform and the extracts were dried by rota-evaporation and resuspended in methanol. Extracts were diluted 1/10 in 0.01% formic acid prior to analysis by LC-MS as described previously [2].

### Cell viability analysis

Following culturing and treatment as described above, mycelia were removed from the culture supernatant and washed with water. They were resuspended in 10 mM HEPES before aliquots were plated on Sabouraud dextrose agar and incubated at 37°C for 24 h. Viability was recorded.

## Electronic supplementary material

Additional file 1: Gene sequencing information. Combined FPKM values for all replicates, Log_2_ fold changes and statistical significance data for all genes. Processed RNA-seq data. Relative expression of genes in *A. fumigatus* +/− gliotoxin; Relative expression of genes in *A. fumigatus* ∆*gliT* +/− gliotoxin. (ZIP 386 KB)

Additional file 2: Additional study information. Additional tables containing the functional categorisation of differentially regulated genes, Illumina RNA-seq summary statistics and primers used in the study. Additional Figure S1 is included. FunCat and KEGG categories of genes altered in expression; Summary Statistics for Illumina RNA-seq; Primers used for qRT-PCR; cell viability data. (DOCX 116 KB)

## Abbreviations

GSH: Glutathione
qRT-PCR: Quantitative real-time PCR
NRPS: Non-ribosomal peptide synthetase
PKS: Polyketide synthase
DTT: Dithiothreitol
IAA: Iodoacetamide.
