# Supplementary material for: RNA-seq reveals the pan-transcriptomic impact of attenuating the gliotoxin self-protection mechanism in Aspergillus fumigatus
Source: BMC Genomics. 2014 Oct 14;15(1):894. doi: 10.1186/1471-2164-15-894 (PMC4209032; doi:10.1186/1471-2164-15-894)
Supplement: Supplementary file 2 — Additional file 2: Additional study information. Additional tables containing the functional categorisation of differentially regulated genes, Illumina RNA-seq summary statistics and primers used in the study. Additional Figure S1 is included. FunCat and KEGG categories of genes altered in expression; Summary Statistics for Illumina RNA-seq; Primers used for qRT-PCR; cell viability data. (DOCX 116 KB) [file 12864_2014_6591_MOESM2_ESM.docx]

Additional Table S1: FunCat Categories of up-regulated genes in ∆*gliT* MeOH v ATCC46645 MeOH

| **FunCat ID** | **FunCat Category** | **No. of genes** | **p-value** |
| --- | --- | --- | --- |
| 01.06.06 | isoprenoid metabolism | 9 | 0.000498 |
| 01.06.06.05 | sesquiterpenes metabolism | 5 | 3.96E-07 |
| 01.06.06.11 | tetracyclic and pentacyclic triterpenes (cholesterin, steroids and hopanoids) metabolism | 5 | 0.02511 |
| 01.01.09.04 | metabolism of phenylalanine | 4 | 0.020364 |
| 1.2 | secondary metabolism | 23 | 1.59E-06 |
| 01.20.05.09 | metabolism of eicosanoids | 2 | 0.01226 |
| 01.20.17.09 | metabolism of alkaloids | 2 | 0.039122 |
| 01.20.33 | metabolism of secondary products derived from L-tryptophan | 2 | 0.02148 |
| 10.03 | cell cycle | 1 | 0.000494 |
| 10.03.01 | mitotic cell cycle and cell cycle control | 1 | 0.009208 |
| 14.07 | protein modification | 4 | 0.014708 |
| 16.01 | protein binding | 4 | 4.61E-07 |
| 16.03 | nucleic acid binding | 2 | 0.001464 |
| 16.21.01 | heme binding | 6 | 0.000882 |
| 20.01 | transported compounds (substrates) | 37 | 1.52E-05 |
| 20.01.01 | ion transport | 11 | 0.001083 |
| 20.01.01.01 | cation transport (H+, Na+, K+, Ca2+ , NH4+, etc.) | 10 | 0.000718 |
| 20.01.01.01.01 | heavy metal ion transport (Cu+, Fe3+, etc.) | 9 | 1.52E-06 |
| 20.01.01.01.01.01 | siderophore-iron transport | 7 | 3.08E-07 |
| 20.01.01.07.07 | phosphate transport | 2 | 0.032776 |
| 20.01.11 | amine / polyamine transport | 4 | 0.015295 |
| 20.01.13 | lipid/fatty acid transport | 5 | 0.040613 |
| 20.01.15 | electron transport | 11 | 0.006083 |
| 20.01.26 | neurotransmitter transport | 3 | 0.021951 |
| 20.01.27 | drug/toxin transport | 9 | 0.00052 |
| 20.03 | transport facilities | 17 | 0.014017 |
| 20.03.02.02 | symporter | 3 | 0.048048 |
| 20.03.25 | ABC transporters | 10 | 9.80E-07 |
| 20.09 | transport routes | 24 | 0.023615 |
| 20.09.16.01 | Type I protein secretion system (ABC-type transport systems) | 5 | 0.000304 |
| 20.09.18 | cellular import | 15 | 0.001044 |
| 32.05 | disease, virulence and defense | 14 | 0.000878 |
| 32.05.01 | resistance proteins | 7 | 0.006809 |
| 32.05.05.01 | toxins | 3 | 0.034548 |
| 32.07 | detoxification | 22 | 3.14E-08 |
| 32.07.01 | detoxification involving cytochrome P450 | 9 | 7.69E-07 |
| 32.07.05 | detoxification by export | 6 | 0.010986 |
| 34.01 | homeostasis | 12 | 0.000705 |
| 34.01.01 | homeostasis of cations | 11 | 0.000707 |
| 34.01.01.01 | homeostasis of metal ions (Na, K, Ca etc.) | 9 | 0.00106 |
| 34.01.03 | homeostasis of anions | 3 | 0.00723 |
| 34.01.03.03 | homeostasis of phosphate | 2 | 0.014356 |
| 41.01 | fungal/microorganismic development | 4 | 0.047098 |
| 41.01.03 | tissue pattern formation | 3 | 0.001399 |
| 41.01.03.01 | fruit body development (sexually or asexually derived spores) | 3 | 0.000928 |
| 43.01 | fungal/microorganismic cell type differentiation | 2 | 0.04479 |
| 43.01.03 | fungal and other eukaryotic cell type differentiation | 2 | 0.04479 |

Additional Table S2: FunCat Categories of down-regulated genes in ∆*gliT* MeOH v ATCC46645 MeOH

| **FunCat ID** | **FunCat Category** | **No. of genes** | **p-value** |
| --- | --- | --- | --- |
| 01.01.06.04.02 | degradation of threonine | 1 | 0.039609 |
| 01.01.09.02.02 | degradation of serine | 1 | 0.043486 |
| 01.02.02 | nitrogen metabolism | 2 | 0.030127 |
| 1.05 | C-compound and carbohydrate metabolism | 10 | 0.044193 |
| 01.05.06 | C-2 compound and organic acid metabolism | 2 | 0.007604 |
| 01.05.06.07 | C-2 compound and organic acid catabolism | 2 | 0.004761 |
| 01.20.17 | metabolism of secondary products derived from primary amino acids | 2 | 0.036082 |
| 01.20.17.03 | metabolism of amines | 2 | 0.006311 |
| 01.25.03 | extracellular protein degradation | 1 | 0.019996 |
| 2.01 | glycolysis and gluconeogenesis | 3 | 0.010523 |
| 02.16.01 | alcohol fermentation | 2 | 0.010002 |
| 16.01 | protein binding | 1 | 0.015303 |
| 16.17.01 | calcium binding | 2 | 0.036082 |
| 16.17.09 | heavy metal binding (Cu, Fe, Zn) | 5 | 0.034355 |
| 16.21.01 | heme binding | 2 | 0.039675 |
| 20.01.01.01.01 | heavy metal ion transport (Cu+, Fe3+, etc.) | 2 | 0.039675 |
| 20.09.18 | cellular import | 5 | 0.025931 |
| 20.09.18.07 | non-vesicular cellular import | 3 | 0.044828 |
| 32.05.03 | defense related proteins | 2 | 0.029312 |

Additional Table S3: KEGG Categories of up-regulated genes in ATCC46645 MeOH v ∆*gliT* MeOH

| **KEGG number** | | **KEGG Pathway** | **No. of genes** | | | **p-value** |
| --- | --- | --- | --- | --- | --- | --- |
| 0.1.2 | Biosynthesis of secondary metabolites | | | 12 | 0.020613 | |
| 1 | Metabolism | | | 27 | 0.005372 | |
| 1.1 | Carbohydrate Metabolism | | | 12 | 0.045957 | |
| 1.1.15 | Inositol phosphate metabolism | | | 2 | 0.03607 | |
| 1.3 | Lipid Metabolism | | | 9 | 0.005247 | |
| 1.3.4 | Synthesis and degradation of ketone bodies | | | 2 | 0.008389 | |
| 1.3.10 | Glycerophospholipid metabolism | | | 3 | 0.021658 | |
| 1.3.11 | Ether lipid metabolism | | | 2 | 0.00659 | |
| 1.8.2 | Riboflavin metabolism | | | 4 | 0.000976 | |
| 1.9 | Metabolism of Terpenoids and Polyketides | | | 8 | 0.000542 | |
| 1.9.9 | Limonene and pinene degradation | | | 7 | 0.001344 | |

Additional Table S4: KEGG Categories of down-regulated genes in ATCC46645 MeOH v ∆*gliT* MeOH

| **KEGG number** | **KEGG Pathway** | **No. of genes** | **p-value** |
| --- | --- | --- | --- |
| 0.1.2 | Biosynthesis of secondary metabolites | 6 | 0.001579 |
| 1.1.1 | Glycolysis / Gluconeogenesis | 3 | 0.001223 |
| 1.1.7 | Ascorbate and aldarate metabolism | 1 | 0.029675 |
| 1.3.3 | Fatty acid metabolism | 2 | 0.007948 |
| 1.5 | Amino Acid Metabolism | 6 | 0.000529 |
| 1.5.2 | Glycine, serine and threonine metabolism | 3 | 0.000979 |
| 1.5.9 | Histidine metabolism | 2 | 0.012132 |
| 1.5.10 | Tyrosine metabolism | 4 | 0.000114 |
| 1.5.11 | Phenylalanine metabolism | 2 | 0.012132 |
| 1.6 | Metabolism of Other Amino Acids | 5 | 3.77E-05 |
| 1.6.1 | beta-Alanine metabolism | 3 | 8.81E-05 |
| 1.11 | Xenobiotics Biodegradation and Metabolism | 2 | 0.004967 |
| 1.11.18 | Metabolism of xenobiotics by cytochrome P450 | 2 | 0.004967 |

Additional Table S5: FunCat Categories of up-regulated genes in ATCC46645 MeOH v ATCC46645 Gliotoxin

| **FunCat ID** | **FunCat Category** | **No. of genes** | **p-value** |
| --- | --- | --- | --- |
| 01.02.07 | regulation of nitrogen, sulfur and selenium metabolism | 3 | 0.017385 |
| 01.02.07.01 | regulation of nitrogen metabolism | 3 | 0.002781 |
| 01.06.06 | isoprenoid metabolism | 5 | 0.033215 |
| 01.06.06.07 | diterpenes metabolism | 2 | 0.004108 |
| 1.2 | secondary metabolism | 16 | 0.000226 |
| 01.20.36 | non-ribosomal peptide synthesis | 2 | 0.020054 |
| 01.20.37 | metabolism of peptide derived compounds | 5 | 0.000183 |
| 01.20.37.01 | metabolism of thioredoxin, glutaredoxin, glutathione | 3 | 0.000781 |
| 01.20.37.03 | metabolism of peptide antibiotics | 2 | 0.020054 |
| 02.16.03.03 | heterofermentative pathway and fermentaton of other saccharides | 1 | 0.021958 |
| 16.01 | protein binding | 7 | 0.003838 |
| 16.17 | metal binding | 2 | 0.020057 |
| 20.01 | transported compounds (substrates) | 29 | 6.53E-05 |
| 20.01.01 | ion transport | 10 | 0.000473 |
| 20.01.01.01 | cation transport (H+, Na+, K+, Ca2+ , NH4+, etc.) | 8 | 0.001876 |
| 20.01.01.01.01 | heavy metal ion transport (Cu+, Fe3+, etc.) | 6 | 0.000211 |
| 20.01.01.01.01.01 | siderophore-iron transport | 5 | 2.61E-05 |
| 20.01.27 | drug/toxin transport | 10 | 1.00E-05 |
| 20.03 | transport facilities | 15 | 0.004936 |
| 20.03.22 | transport ATPases | 7 | 9.55E-05 |
| 20.03.25 | ABC transporters | 7 | 8.38E-05 |
| 20.09 | transport routes | 20 | 0.015186 |
| 20.09.16 | cellular export and secretion | 9 | 0.001148 |
| 20.09.16.01 | Type I protein secretion system (ABC-type transport systems) | 4 | 0.001102 |
| 20.09.18 | cellular import | 11 | 0.006834 |
| 20.09.18.07 | non-vesicular cellular import | 7 | 0.006127 |
| 32.05 | disease, virulence and defense | 11 | 0.002563 |
| 32.05.01 | resistance proteins | 6 | 0.007123 |
| 32.05.03 | defense related proteins | 4 | 0.006148 |
| 32.07 | Detoxification | 18 | 2.04E-07 |
| 32.07.01 | detoxification involving cytochrome P450 | 3 | 0.046189 |
| 32.07.05 | detoxification by export | 7 | 0.000543 |
| 32.07.07 | oxygen and radical detoxification | 4 | 0.005223 |
| 32.07.07.03 | glutathione conjugation reaction | 2 | 0.018176 |
| 34.01 | Homeostasis | 10 | 0.001026 |
| 34.01.01 | homeostasis of cations | 8 | 0.005279 |
| 34.01.01.01 | homeostasis of metal ions (Na, K, Ca etc.) | 8 | 0.000763 |
| 34.01.03 | homeostasis of anions | 2 | 0.037714 |
| 34.01.03.01 | homeostasis of sulfate | 1 | 0.043442 |

Additional Table S6: FunCat Categories of down-regulated genes in ATCC46645 MeOH v ATCC46645 Gliotoxin

| **FunCat ID** | **FunCat Category** | **No. of genes** | **p-value** |
| --- | --- | --- | --- |
| 1.01 | amino acid metabolism | 13 | 0.000154 |
| 01.01.03 | assimilation of ammonia, metabolism of the glutamate group | 3 | 0.044683 |
| 01.01.03.05 | metabolism of arginine | 2 | 0.034001 |
| 01.01.05 | metabolism of urea cycle, creatine and polyamines | 2 | 0.042884 |
| 01.01.05.01 | metabolism of polyamines | 2 | 0.008585 |
| 01.01.09 | metabolism of the cysteine - aromatic group | 6 | 0.005937 |
| 01.01.09.04 | metabolism of phenylalanine | 3 | 0.019408 |
| 01.01.09.05 | metabolism of tyrosine | 3 | 0.006186 |
| 1.02 | nitrogen, sulfur and selenium metabolism | 8 | 0.001192 |
| 01.02.02 | nitrogen metabolism | 4 | 0.001902 |
| 1.05 | C-compound and carbohydrate metabolism | 17 | 0.032987 |
| 01.05.02.04 | sugar, glucoside, polyol and carboxylate anabolism | 3 | 0.037325 |
| 01.05.06 | C-2 compound and organic acid metabolism | 2 | 0.027495 |
| 01.05.06.07 | C-2 compound and organic acid catabolism | 2 | 0.017523 |
| 01.05.10 | aliphatic hydrocarbon metabolism | 1 | 0.038822 |
| 01.05.10.07 | aliphatic hydrocarbon catabolism | 1 | 0.038822 |
| 01.20.17 | metabolism of secondary products derived from primary amino acids | 3 | 0.020843 |
| 01.20.17.03 | metabolism of amines | 3 | 0.001603 |
| 2.01 | glycolysis and gluconeogenesis | 6 | 0.000252 |
| 02.01.01 | glycolysis methylglyoxal bypass | 1 | 0.046407 |
| 2.08 | pyruvate dehydrogenase complex | 2 | 0.002628 |
| 2.13 | respiration | 4 | 0.032922 |
| 02.13.03 | aerobic respiration | 3 | 0.031565 |
| 2.16 | fermentation | 4 | 0.005473 |
| 02.16.01 | alcohol fermentation | 3 | 0.003187 |
| 02.16.09 | mixed acid and butanediol fermentation | 1 | 0.015709 |
| 10.03 | cell cycle | 1 | 0.037294 |
| 16.01 | protein binding | 5 | 0.017031 |
| 16.17 | metal binding | 14 | 0.000681 |
| 16.17.01 | calcium binding | 4 | 0.002727 |
| 16.17.09 | heavy metal binding (Cu, Fe, Zn) | 12 | 0.000173 |
| 16.21 | complex cofactor/cosubstrate/vitamine binding | 12 | 0.000107 |
| 16.21.01 | heme binding | 4 | 0.003295 |
| 20.01.15 | electron transport | 8 | 0.003303 |
| 20.01.27 | drug/toxin transport | 4 | 0.0393 |
| 20.03.22 | transport ATPases | 3 | 0.042512 |
| 20.09 | transport routes | 3 | 0.034473 |

Additional Table S7: FunCat Categories of up-regulated genes in *A. fumigatus* ∆*gliT* MeOH v *A. fumigatus* ∆*gliT* Gliotoxin

| **FunCat ID** | **FunCat Category** | **No. of genes** | **p-value** |
| --- | --- | --- | --- |
| 01.01.06 | metabolism of the aspartate family | 21 | 4.34E-05 |
| 01.01.06.04 | metabolism of threonine | 4 | 0.016958 |
| 01.01.06.05 | metabolism of methionine | 16 | 8.06E-09 |
| 01.01.09.01 | metabolism of glycine | 6 | 0.023326 |
| 01.01.09.02 | metabolism of serine | 7 | 0.019309 |
| 01.01.09.03 | metabolism of cysteine | 5 | 0.017006 |
| 01.02.03 | sulfur metabolism | 4 | 0.010337 |
| 01.02.03.01 | sulfate assimilation | 4 | 0.002668 |
| 01.03.01.03 | purine nucleotide/nucleoside/nucleobase anabolism | 5 | 0.046996 |
| 01.03.16 | polynucleotide degradation | 2 | 0.047423 |
| 1.04 | phosphate metabolism | 25 | 0.02562 |
| 1.05 | C-compound and carbohydrate metabolism | 55 | 2.68E-05 |
| 01.05.02 | sugar, glucoside, polyol and carboxylate metabolism | 8 | 0.002658 |
| 01.05.02.07 | sugar, glucoside, polyol and carboxylate catabolism | 5 | 0.007245 |
| 01.05.03 | polysaccharide metabolism | 4 | 0.000483 |
| 01.05.13.03 | tetrahydrofolate-dependent C-1-transfer | 4 | 0.025738 |
| 1.06 | lipid, fatty acid and isoprenoid metabolism | 32 | 0.008308 |
| 01.06.06 | isoprenoid metabolism | 4 | 0.03403 |
| 1.2 | secondary metabolism | 22 | 0.010633 |
| 12.01 | ribosome biogenesis | 105 | 1.28E-61 |
| 12.01.01 | ribosomal proteins | 98 | 3.40E-77 |
| 12.04 | translation | 86 | 8.18E-49 |
| 12.04.02 | translation elongation | 5 | 0.027556 |
| 12.07 | translational control | 10 | 0.039614 |
| 14.04 | protein targeting, sorting and translocation | 11 | 0.040203 |
| 14.07 | protein modification | 34 | 0.007761 |
| 14.07.03 | modification by phosphorylation, dephosphorylation, autophosphorylation | 6 | 0.00518 |
| 16.01 | protein binding | 146 | 1.69E-08 |
| 16.03 | nucleic acid binding | 66 | 0.00865 |
| 16.03.03 | RNA binding | 39 | 3.95E-05 |
| 16.17 | metal binding | 33 | 0.040594 |
| 16.19.05 | GTP binding | 2 | 0.034046 |
| 18.01 | regulation by | 7 | 0.006734 |
| 18.01.01 | regulation by modification | 3 | 0.012856 |
| 18.02 | regulation of protein activity | 19 | 0.009861 |
| 20.01 | transported compounds (substrates) | 60 | 0.000723 |
| 20.09 | transport routes | 46 | 0.000475 |
| 20.09.01 | nuclear transport | 1 | 0.042961 |
| 20.09.07 | vesicular transport (Golgi network, etc.) | 5 | 0.002029 |
| 20.09.13 | vacuolar/lysosomal transport | 1 | 0.000691 |
| 20.09.18.09 | vesicular cellular import | 2 | 0.033669 |
| 30.01 | cellular signalling | 14 | 0.000329 |
| 30.01.05 | enzyme mediated signal transduction | 6 | 0.000806 |
| 30.01.05.01 | protein kinase | 3 | 0.024907 |
| 30.01.09 | second messenger mediated signal transduction | 1 | 0.030343 |
| 32.01 | stress response | 29 | 0.039761 |
| 32.01.01 | oxidative stress response | 2 | 0.046596 |
| 32.01.07 | unfolded protein response (e.g. ER quality control) | 10 | 0.033292 |
| 32.05 | disease, virulence and defense | 13 | 0.009417 |
| 32.07 | detoxification | 14 | 0.008595 |
| 34.11.03 | chemoperception and response | 2 | 0.008362 |
| 40.01 | cell growth / morphogenesis | 8 | 0.000427 |
| 40.01.03 | directional cell growth (morphogenesis) | 1 | 0.009629 |
| 42.01 | cell wall | 7 | 0.009139 |
| 42.04.03 | actin cytoskeleton | 2 | 0.047004 |
| 42.16 | mitochondrion | 27 | 9.96E-07 |
| 43.01 | fungal/microorganismic cell type differentiation | 15 | 0.000955 |
| 43.01.03 | fungal and other eukaryotic cell type differentiation | 15 | 0.000955 |
| 43.01.03.05 | budding, cell polarity and filament formation | 9 | 0.039187 |

Additional Table S8: FunCat Categories of down-regulated genes *A. fumigatus* ∆*gliT* MeOH v *A. fumigatus* ∆*gliT* Gliotoxin

| **FunCat ID** | **FunCat Category** | **No. of genes** | **p-value** |
| --- | --- | --- | --- |
| 1.01 | amino acid metabolism | 93 | 5.67E-05 |
| 01.01.03.03 | metabolism of proline | 5 | 0.042464 |
| 01.01.05.01 | metabolism of polyamines | 6 | 0.014408 |
| 01.01.09 | metabolism of the cysteine - aromatic group | 35 | 0.039198 |
| 01.01.09.04 | metabolism of phenylalanine | 16 | 0.015763 |
| 01.01.11 | metabolism of the pyruvate family (alanine, isoleucine, leucine, valine) and D-alanine | 15 | 0.008954 |
| 01.01.11.04 | metabolism of leucine | 10 | 0.007366 |
| 1.02 | nitrogen, sulfur and selenium metabolism | 57 | 1.44E-05 |
| 01.02.02 | nitrogen metabolism | 17 | 0.002115 |
| 01.02.02.09 | catabolism of nitrogenous compounds | 8 | 0.024727 |
| 1.03 | nucleotide/nucleoside/nucleobase metabolism | 28 | 0.006103 |
| 01.03.16 | polynucleotide degradation | 5 | 0.015934 |
| 1.04 | phosphate metabolism | 52 | 0.029279 |
| 1.05 | C-compound and carbohydrate metabolism | 211 | 1.68E-06 |
| 01.05.02 | sugar, glucoside, polyol and carboxylate metabolism | 50 | 0.017509 |
| 01.05.02.07 | sugar, glucoside, polyol and carboxylate catabolism | 40 | 0.00538 |
| 01.05.03 | polysaccharide metabolism | 45 | 0.003302 |
| 01.05.03.01 | glycogen metabolism | 4 | 0.005114 |
| 01.05.06 | C-2 compound and organic acid metabolism | 12 | 0.000234 |
| 01.05.06.07 | C-2 compound and organic acid catabolism | 10 | 0.000463 |
| 01.05.08 | C-4 compound metabolism | 3 | 0.01382 |
| 01.05.09.07 | aminosaccharide catabolism | 7 | 0.027922 |
| 01.05.10 | aliphatic hydrocarbon metabolism | 3 | 0.01382 |
| 01.05.10.07 | aliphatic hydrocarbon catabolism | 3 | 0.01382 |
| 01.05.11 | aromate metabolism | 15 | 0.027907 |
| 01.05.11.07 | aromate catabolism | 11 | 0.049486 |
| 1.06 | lipid, fatty acid and isoprenoid metabolism | 119 | 0.000332 |
| 01.06.05 | fatty acid metabolism | 29 | 0.004169 |
| 01.06.06.05 | sesquiterpenes metabolism | 6 | 0.001339 |
| 01.08.02 | metabolism of hormones | 3 | 0.006062 |
| 1.2 | secondary metabolism | 103 | 1.01E-06 |
| 01.20.05.09 | metabolism of eicosanoids | 4 | 0.044562 |
| 01.20.15.01 | metabolism of naphthoquinone, anthraquinone | 2 | 0.038836 |
| 01.20.36 | non-ribosomal peptide synthesis | 6 | 0.02449 |
| 2.01 | glycolysis and gluconeogenesis | 23 | 0.012161 |
| 02.01.01 | glycolysis methylglyoxal bypass | 4 | 0.002418 |
| 02.11.05 | accessory proteins of electron transport and membrane-associated energy conservation | 1 | 0.025535 |
| 02.13.03 | aerobic respiration | 4 | 0.029733 |
| 2.16 | fermentation | 21 | 0.002643 |
| 02.16.01 | alcohol fermentation | 13 | 0.000262 |
| 2.19 | metabolism of energy reserves (e.g. glycogen, trehalose) | 14 | 0.032019 |
| 10.01 | DNA processing | 24 | 2.49E-11 |
| 10.01.02 | DNA topology | 2 | 0.002266 |
| 10.01.03 | DNA synthesis and replication | 3 | 1.56E-05 |
| 10.01.05 | DNA recombination and DNA repair | 8 | 9.67E-09 |
| 10.01.05.01 | DNA repair | 7 | 3.27E-07 |
| 10.01.05.03 | DNA recombination | 3 | 0.000212 |
| 10.01.09 | DNA restriction or modification | 15 | 0.000154 |
| 10.01.09.05 | DNA conformation modification (e.g. chromatin) | 15 | 0.000275 |
| 10.03 | cell cycle | 32 | 6.83E-12 |
| 10.03.01 | mitotic cell cycle and cell cycle control | 23 | 5.44E-09 |
| 10.03.01.01 | mitotic cell cycle | 9 | 9.66E-05 |
| 10.03.01.03 | cell cycle checkpoints (checkpoints of morphogenesis, DNA-damage,-replication, mitotic phase and spindle) | 3 | 0.021885 |
| 10.03.03 | cytokinesis (cell division) /septum formation and hydrolysis | 5 | 0.000106 |
| 10.03.04 | nuclear and chromosomal cycle | 2 | 0.000108 |
| 10.03.04.05 | chromosome segregation/division | 2 | 0.020358 |
| 10.03.05 | cell cycle dependent cytoskeleton reorganization | 1 | 0.003755 |
| 10.03.05.01 | spindle pole body/centrosome and microtubule cycle | 1 | 0.026401 |
| 11.02 | RNA synthesis | 51 | 2.59E-10 |
| 11.02.03 | mRNA synthesis | 50 | 2.32E-08 |
| 11.02.03.01 | general transcription activities | 9 | 0.002961 |
| 11.02.03.04 | transcriptional control | 50 | 4.00E-05 |
| 11.04 | RNA processing | 9 | 3.40E-14 |
| 11.04.01 | rRNA processing | 2 | 2.61E-08 |
| 11.04.02 | tRNA processing | 2 | 0.041973 |
| 11.04.03 | mRNA processing (splicing, 5'-, 3'-end processing) | 6 | 2.72E-08 |
| 11.04.03.01 | splicing | 4 | 4.64E-07 |
| 11.06 | RNA modification | 2 | 0.020358 |
| 11.06.02 | tRNA modification | 1 | 0.03752 |
| 12.01 | ribosome biogenesis | 2 | 6.10E-12 |
| 12.01.01 | ribosomal proteins | 1 | 2.67E-08 |
| 12.04 | translation | 5 | 4.29E-07 |
| 12.04.01 | translation initiation | 2 | 0.029035 |
| 14.01 | protein folding and stabilization | 5 | 0.001611 |
| 14.1 | assembly of protein complexes | 5 | 2.07E-08 |
| 14.04 | protein targeting, sorting and translocation | 9 | 3.55E-08 |
| 14.07 | protein modification | 70 | 0.006125 |
| 14.07.01 | modification with fatty acids (e.g. myristylation, palmitylation, farnesylation) | 1 | 0.039313 |
| 14.07.04 | modification by acetylation, deacetylation | 2 | 0.014199 |
| 14.07.05 | modification by ubiquitination, deubiquitination | 7 | 0.005774 |
| 14.07.07 | modification by ubiquitin-related proteins | 1 | 0.037562 |
| 14.13.01.01 | proteasomal degradation (ubiquitin/proteasomal pathway) | 6 | 0.000162 |
| 14.13.04 | lysosomal and vacuolar protein degradation | 17 | 6.11E-07 |
| 14.13.04.02 | vacuolar protein degradation | 11 | 1.86E-06 |
| 16.01 | protein binding | 78 | 8.24E-24 |
| 16.03 | nucleic acid binding | 32 | 1.81E-14 |
| 16.03.01 | DNA binding | 24 | 2.58E-06 |
| 16.03.03 | RNA binding | 6 | 8.12E-11 |
| 16.07 | structural protein binding | 2 | 0.041317 |
| 16.17 | metal binding | 100 | 0.039709 |
| 16.17.05 | sodium binding | 4 | 0.022913 |
| 16.17.09 | heavy metal binding (Cu, Fe, Zn) | 76 | 0.002646 |
| 16.19 | nucleotide/nucleoside/nucleobase binding | 48 | 5.41E-09 |
| 16.19.03 | ATP binding | 39 | 3.07E-06 |
| 16.19.05 | GTP binding | 5 | 0.008471 |
| 16.21 | complex cofactor/cosubstrate/vitamine binding | 86 | 1.60E-06 |
| 16.21.01 | heme binding | 25 | 2.20E-06 |
| 18.01 | regulation by | 18 | 0.008169 |
| 18.01.01 | regulation by modification | 9 | 0.016078 |
| 18.02 | regulation of protein activity | 35 | 0.000273 |
| 18.02.01 | enzymatic activity regulation / enzyme regulator | 26 | 0.047686 |
| 18.02.01.01 | enzyme activator | 5 | 0.002299 |
| 20.01 | transported compounds (substrates) | 220 | 5.20E-09 |
| 20.01.01 | ion transport | 49 | 0.000752 |
| 20.01.01.01 | cation transport (H+, Na+, K+, Ca2+ , NH4+, etc.) | 46 | 2.44E-05 |
| 20.01.01.01.01 | heavy metal ion transport (Cu+, Fe3+, etc.) | 22 | 0.000125 |
| 20.01.01.01.01.01 | siderophore-iron transport | 12 | 0.000234 |
| 20.01.03 | C-compound and carbohydrate transport | 42 | 0.015267 |
| 20.01.07 | amino acid/amino acid derivatives transport | 23 | 0.001485 |
| 20.01.09 | peptide transport | 6 | 0.02449 |
| 20.01.10 | protein transport | 7 | 1.36E-06 |
| 20.01.11 | amine / polyamine transport | 24 | 2.69E-07 |
| 20.01.13 | lipid/fatty acid transport | 27 | 0.003886 |
| 20.01.15 | electron transport | 69 | 5.13E-07 |
| 20.01.17 | nucleotide/nucleoside/nucleobase transport | 7 | 0.033968 |
| 20.01.23 | allantoin and allantoate transport | 11 | 0.046105 |
| 20.01.25 | vitamine/cofactor transport | 16 | 0.003346 |
| 20.01.26 | neurotransmitter transport | 15 | 5.02E-05 |
| 20.01.27 | drug/toxin transport | 42 | 8.60E-07 |
| 20.03 | transport facilities | 121 | 2.50E-07 |
| 20.03.02 | carrier (electrochemical potential-driven transport) | 35 | 9.89E-07 |
| 20.03.02.02 | symporter | 15 | 0.002761 |
| 20.03.02.03 | antiporter | 21 | 5.64E-05 |
| 20.03.25 | ABC transporters | 19 | 0.025255 |
| 20.09.01 | nuclear transport | 1 | 0.000516 |
| 20.09.04 | mitochondrial transport | 4 | 0.004171 |
| 20.09.07 | vesicular transport (Golgi network, etc.) | 9 | 6.86E-06 |
| 20.09.18 | cellular import | 78 | 9.66E-05 |
| 20.09.18.07 | non-vesicular cellular import | 35 | 0.018662 |
| 30.01 | cellular signalling | 30 | 1.85E-05 |
| 30.01.05 | enzyme mediated signal transduction | 14 | 3.61E-05 |
| 30.01.05.01 | protein kinase | 8 | 0.013121 |
| 30.01.05.05 | G-protein mediated signal transduction | 2 | 5.00E-05 |
| 32.01.04 | pH stress response | 4 | 0.015146 |
| 32.01.07 | unfolded protein response (e.g. ER quality control) | 3 | 0.02153 |
| 32.01.09 | DNA damage response | 7 | 0.014162 |
| 32.05 | disease, virulence and defense | 85 | 1.74E-09 |
| 32.05.01 | resistance proteins | 36 | 7.17E-05 |
| 32.05.03 | defense related proteins | 14 | 0.033645 |
| 32.05.05 | virulence, disease factors | 32 | 0.000761 |
| 32.05.05.01 | toxins | 13 | 0.006216 |
| 32.07 | detoxification | 90 | 1.31E-09 |
| 32.07.01 | detoxification involving cytochrome P450 | 22 | 2.14E-05 |
| 32.07.03 | detoxification by modification | 11 | 0.041021 |
| 32.07.05 | detoxification by export | 29 | 0.000841 |
| 34.01 | homeostasis | 61 | 3.12E-06 |
| 34.01.01 | homeostasis of cations | 56 | 8.46E-07 |
| 34.01.01.01 | homeostasis of metal ions (Na, K, Ca etc.) | 42 | 1.17E-05 |
| 34.01.03 | homeostasis of anions | 7 | 0.040824 |
| 34.11 | cellular sensing and response to external stimulus | 47 | 0.026046 |
| 34.11.13 | gas and metabolite distribution | 4 | 0.044562 |
| 34.11.13.07 | circulation (e.g. blood pressure) | 4 | 0.032698 |
| 40.01 | cell growth / morphogenesis | 27 | 0.012945 |
| 40.02 |  | 4 | 0.022913 |
| 40.02.03 | activity of intercellular mediators | 4 | 0.022913 |
| 40.10.02.01 | anti-apoptosis | 8 | 0.010649 |
| 41.01.03 | tissue pattern formation | 6 | 0.007644 |
| 41.01.03.01 | fruit body development (sexually or asexually derived spores) | 6 | 0.00353 |
| 42.1 | nucleus | 15 | 3.49E-05 |
| 42.04 | cytoskeleton/structural proteins | 10 | 1.33E-06 |
| 42.04.03 | actin cytoskeleton | 4 | 0.004171 |
| 42.04.05 | microtubule cytoskeleton | 2 | 0.004758 |
| 42.10.03 | organization of chromosome structure | 15 | 0.00084 |
| 42.27 | extracellular / secretion proteins | 6 | 0.046962 |
| 43.01 | fungal/microorganismic cell type differentiation | 40 | 0.00999 |
| 43.01.03 | fungal and other eukaryotic cell type differentiation | 40 | 0.00999 |
| 43.01.03.05 | budding, cell polarity and filament formation | 15 | 0.00084 |

Additional Table S9: KEGG Categories of up-regulated genes in ATCC46645 MeOH v ATCC46645 Gliotoxin

| **KEGG number** | **KEGG Pathway** | **No. of genes** | **p-value** |
| --- | --- | --- | --- |
| 1.11 | Xenobiotics Biodegradation and Metabolism | 2 | 0.011312 |
| 1.11.18 | Metabolism of xenobiotics by cytochrome P450 | 2 | 0.011312 |

Additional Table S10: KEGG Categories of down-regulated genes in ATCC46645 MeOH v ATCC46645 Gliotoxin

| **KEGG number** | **KEGG Pathway** | **No. of genes** | **p-value** |
| --- | --- | --- | --- |
| 0.1.1 | Metabolic pathways | 19 | 6.48E-05 |
| 0.1.2 | Biosynthesis of secondary metabolites | 12 | 0.00029 |
| 1 | Metabolism | 20 | 0.003936 |
| 1.1.1 | Glycolysis / Gluconeogenesis | 3 | 0.022129 |
| 1.1.7 | Ascorbate and aldarate metabolism | 2 | 0.00245 |
| 1.5 | Amino Acid Metabolism | 17 | 2.39E-10 |
| 1.5.2 | Glycine, serine and threonine metabolism | 4 | 0.002102 |
| 1.5.4 | Valine, leucine and isoleucine degradation | 4 | 0.000834 |
| 1.5.8 | Arginine and proline metabolism | 3 | 0.017141 |
| 1.5.10 | Tyrosine metabolism | 9 | 1.80E-08 |
| 1.5.11 | Phenylalanine metabolism | 6 | 3.29E-06 |
| 1.6 | Metabolism of Other Amino Acids | 4 | 0.035241 |
| 1.6.1 | beta-Alanine metabolism | 4 | 9.15E-05 |

Additional Table S11: KEGG Categories of up-regulated genes in *A. fumigatus* ∆*gliT* MeOH v *A. fumigatus* ∆*gliT* gliotoxin

| **KEGG number** | **KEGG Pathway** | **No. of genes** | **p-value** |
| --- | --- | --- | --- |
| 0.1.1 | Metabolic pathways | 58 | 6.74E-05 |
| 1 | Metabolism | 75 | 8.25E-12 |
| 1.1 | Carbohydrate Metabolism | 20 | 4.67E-05 |
| 1.1.9 | Amino sugar and nucleotide sugar metabolism | 2 | 0.028819 |
| 1.2.9 | Sulfur metabolism | 7 | 0.000198 |
| 1.3 | Lipid Metabolism | 8 | 0.001172 |
| 1.5.3 | Cysteine and methionine metabolism | 12 | 2.08E-05 |
| 1.6.4 | Selenoamino acid metabolism | 9 | 0.001556 |
| 1.7 | Glycan Biosynthesis and Metabolism | 2 | 0.042004 |
| 1.8.3 | Vitamin B6 metabolism | 3 | 0.021708 |
| 1.8.9 | One carbon pool by folate | 4 | 0.036031 |
| 1.9 | Metabolism of Terpenoids and Polyketides | 3 | 0.002402 |
| 1.9.9 | Limonene and pinene degradation | 3 | 0.010487 |
| 2 | Genetic Information Processing | 94 | 7.13E-17 |
| 2.2.1 | Ribosome | 67 | 3.30E-68 |
| 2.2 | Translation | 70 | 2.55E-49 |
| 4 | Cellular Processes | 8 | 0.026965 |
| 4.1 | Transport and Catabolism | 1 | 0.002435 |

Additional Table S12: KEGG Categories of down-regulated genes in *A. fumigatus* ∆*gliT* MeOH v *A. fumigatus* ∆*gliT* gliotoxin

| **KEGG number** | **KEGG Pathway** | **No. of genes** | **p-value** |
| --- | --- | --- | --- |
| 0.1.1 | Metabolic pathways | 124 | 0.000226 |
| 0.1.2 | Biosynthesis of secondary metabolites | 62 | 0.001578 |
| 1 | Metabolism | 186 | 3.55E-15 |
| 1.1 | Carbohydrate Metabolism | 84 | 5.28E-09 |
| 1.1 | Biosynthesis of Other Secondary Metabolites | 5 | 9.21E-05 |
| 1.1.1 | Glycolysis / Gluconeogenesis | 11 | 0.047858 |
| 1.1.8 | Starch and sucrose metabolism | 15 | 0.027631 |
| 1.1.11 | Glyoxylate and dicarboxylate metabolism | 5 | 0.042527 |
| 1.1.12 | Propanoate metabolism | 13 | 0.00066 |
| 1.1.13 | Butanoate metabolism | 20 | 0.00198 |
| 1.2.1 | Oxidative phosphorylation | 2 | 0.013538 |
| 1.3 | Lipid Metabolism | 40 | 0.000795 |
| 1.3.3 | Fatty acid metabolism | 9 | 0.008109 |
| 1.3.4 | Synthesis and degradation of ketone bodies | 4 | 0.012232 |
| 1.4.2 | Pyrimidine metabolism | 2 | 0.039747 |
| 1.5 | Amino Acid Metabolism | 57 | 0.000136 |
| 1.5.4 | Valine, leucine and isoleucine degradation | 13 | 0.000231 |
| 1.5.10 | Tyrosine metabolism | 18 | 0.000628 |
| 1.5.11 | Phenylalanine metabolism | 10 | 0.01954 |
| 1.5.12 | Tryptophan metabolism | 10 | 0.005708 |
| 1.6 | Metabolism of Other Amino Acids | 21 | 0.020068 |
| 1.6.1 | beta-Alanine metabolism | 7 | 0.010189 |
| 1.6.5 | Cyanoamino acid metabolism | 7 | 0.020421 |
| 1.6.7 | D-Arginine and D-ornithine metabolism | 2 | 0.0347 |
| 1.7.15 | Other glycan degradation | 4 | 0.048468 |
| 1.8.2 | Riboflavin metabolism | 8 | 0.011536 |
| 1.9 | Metabolism of Terpenoids and Polyketides | 23 | 0.012106 |
| 1.9.9 | Limonene and pinene degradation | 22 | 0.004694 |
| 1.10.11 | Caffeine metabolism | 2 | 0.012492 |
| 1.10.15 | Penicillin and cephalosporin biosynthesis | 3 | 0.005089 |
| 2 | Genetic Information Processing | 6 | 2.77E-20 |
| 2.1 | Transcription | 1 | 3.89E-06 |
| 2.2 | Translation | 1 | 3.37E-05 |
| 2.3 | Folding, Sorting and Degradation | 2 | 7.48E-07 |
| 2.4 | Replication and Repair | 2 | 0.004471 |
| 4 | Cellular Processes | 9 | 0.007314 |
| 4.3 | Cell Growth and Death | 1 | 0.000981 |
| 4.3.2 | Cell cycle - yeast | 1 | 0.005251 |

Additional Table S13: Summary statistics of the illumina RNA-Seq. The number of mapped reads is computed from the TopHat alignments to the CADRE 3a *Aspergillus fumigatus* Af293 reference genome.

| Sample Name | Accession | Total Reads | Mapped Reads | Rate |
| --- | --- | --- | --- | --- |
| *A. fumigatus* wild-type_MeOH_Dup1 | ERS132604 | 24504538 | 22855107 | 0.93 |
| *A. fumigatus* wild-type_MeOH_Dup2 | ERS132605 | 21976034 | 20477250 | 0.93 |
| *A. fumigatus* wild-type_MeOH_Dup3 | ERS149278 | 20072230 | 19089952 | 0.95 |
| *A. fumigatus* wild-type_gliotoxin_Dup1 | ERS132606 | 20480420 | 19279500 | 0.94 |
| *A. fumigatus* wild-type_gliotoxin_Dup2 | ERS132607 | 18348804 | 17334037 | 0.94 |
| *A. fumigatus* wild-type_gliotoxin_Dup3 | ERS149279 | 20327620 | 19328531 | 0.95 |
| *A. fumigatus* ∆*gliT*_MeOH_Dup1 | ERS132608 | 19801840 | 18713743 | 0.95 |
| *A. fumigatus* ∆*gliT*_MeOH_Dup2 | ERS132609 | 3636850 | 3438830 | 0.95 |
| *A. fumigatus* ∆*gliT*_MeOH_Dup3 | ERS149280 | 19494792 | 18529853 | 0.95 |
| *A. fumigatus* ∆*gliT*_gliotoxin_Dup1 | ERS132610 | 20170690 | 19206707 | 0.95 |
| *A. fumigatus* ∆*gliT*_gliotoxin_Dup2 | ERS132611 | 18974974 | 17964194 | 0.95 |

Additional Table S14: Primers used for qRT-PCR analysis

| Primer Name | Primer Sequence | Reference |
| --- | --- | --- |
| ogliZ_F | CTCCGATTCCGACCTTTCTT | This study |
| ogliZ_R | GTTAGGCTGCTGAGGGTCTG | This study |
| ogliA_F | GTATGGGTCCGCCTTCTTTC | This study |
| ogliA_R | TGTAGGAGCCCGAGGAGAT | This study |
| oosc3_F | AATTCTGGCTGTGCGTTCTC | This study |
| oosc3_R | CTGCTGATAGGGGATGTCGT | This study |
| olaeA_F | AGGCCGCTCAAGAAACAAC | This study |
| olaeA_R | TCGTCCGTTCTCCTGATAGC | This study |
| ogliT_F | CCGAGTCCGTCACCATCTAC | This study |
| ogliT_R | GCCAAAGATCCCATCGACAC | This study |
| oAFUA_3G13700_F | TTGTTTTCGGAACTGGGAAG | This study |
| oAFUA_3G13700_R | GTTGGCCTGTTGGACTGTTT | This study |
| oftmA_F | CTGCCTGGTTCTAGCCGCAAGG | (Cramer et al., 2006) |
| oftmA_R | TGCGGAGGTATTCCAGCGCAGT | (Cramer et al., 2006) |
| opsoA_F | TGGTGGCGGCCTGATCTACCTT | (Cramer et al., 2006) |
| opsoA_R | TGCCCGAATCACTCCCTTGGAC | (Cramer et al., 2006) |
| ooptB_F | TGTCCTTCTGGCTCGCAGGT | (Hartmann et al., 2011) |
| ooptB_R | ATCCACCATCCGCGGAAACG | (Hartmann et al., 2011) |
| ofma-PKS_F | ATCATCGGATCGGTGAAGAG | This study |
| ofma-PKS_R | GCTGGATTTGGGTTGAGAAA | This study |
| osidH_F | CCTCACATTCTCCTCCTCACA | This study |
| osidH_R | CGTTGAGGTCGTTCCATTCT | This study |

**Figure S1:** Viability assay. There was 85 % survival of ∆*gliT* following gliotoxin treatment compared to 97 % survival in the control group. There was 100 % survival of wild-type both with and without gliotoxin treatment.

**References**

Cramer, R. A, Stajich, J. E., Yamanaka, Y., Dietrich, F. S., Steinbach, W. J., & Perfect, J. R. (2006). Phylogenomic analysis of non-ribosomal peptide synthetases in the genus Aspergillus. *Gene*, *383*, 24–32. doi:10.1016/j.gene.2006.07.008

Hartmann, T., Cairns, T. C., Olbermann, P., Morschhäuser, J., Bignell, E. M., & Krappmann, S. (2011). Oligopeptide transport and regulation of extracellular proteolysis are required for growth of Aspergillus fumigatus on complex substrates but not for virulence. *Molecular Microbiology*, *82*(4), 917–35. doi:10.1111/j.1365-2958.2011.07868.x
